# CBL ubiquitin ligase targets translation as a degrader E3

**DOI:** 10.1039/d5sc06141e

**Published:** 2025-12-26

**Authors:** Alice T. Wicks, Lori Buetow, Toshiyasu Suzuki, Tobias Schmidt, Sergio Lilla, Abigail Macmillan-Jones, Jennifer Turney, Andrea Gohlke, Martin Bushell, Andreas K. Hock, Danny T. Huang

**Affiliations:** a Cancer Research UK Scotland Institute Garscube Estate, Switchback Road Glasgow G61 1BD UK d.huang@crukscotlandinstitute.ac.uk; b School of Cancer Sciences, University of Glasgow Glasgow G61 1QH UK; c AstraZeneca, R&D Biopharmaceuticals, Discovery Sciences, Cellular Assay Development, The Discovery Centre 1 Francis Crick Avenue Cambridge CB2 0AA UK; d AstraZeneca, R&D Biopharmaceuticals, Discovery Sciences, Protein Sciences, Structure & Biophysics, The Discovery Centre 1 Francis Crick Avenue Cambridge CB2 0AA UK

## Abstract

Proteolysis Targeting Chimeras (PROTACs) are heterobifunctional molecules that recruit an ubiquitin ligase (E3) and a neo-substrate into a ternary complex, enabling selective protein degradation. Despite the presence of over 600 E3s, only a handful are utilised in PROTAC application, potentially limiting the number of druggable targets. Here, we investigate whether Casitas B-cell lymphoma (CBL) can be harnessed as a degrader E3 to promote ubiquitination and degradation of the eukaryotic translation initiation factor 4E (eIF4E). Using a selective CBL binding peptide, CBLock, we demonstrate that CBL facilitates the ubiquitination of CBLock-eIF4E fusion in cells and in *in vitro* reconstituted assays. We further developed peptidic PROTACs, termed eIFTerminators, by linking CBLock to an eIF4E-binding peptide. Among them, eIFTerminator4 rapidly eliminates endogenous eIF4E *via* both lysosomal and proteasomal pathways. Unexpectedly, eIFTerminator4 also caused a decrease in eIF4A and eIF4G levels, leading to a reduction in overall protein translation in cells. Our findings establish proof-of-concept that CBL can function as a degrader E3, expanding the arsenal of E3s available for targeted protein degradation in combating challenging drug targets.

## Abstract 2

The peptidic PROTAC elFTerminator brings CBL and elF4E together and promotes CBL-mediated elF4E degradation. Consequently, protein translation is disrupted.

## Graphical abstract

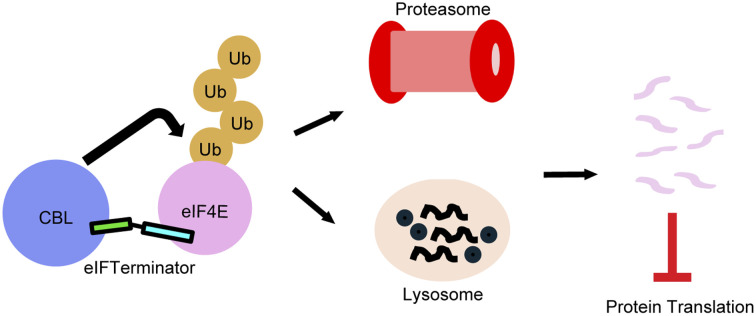

## Introduction

Proteins are constantly degraded and replenished to maintain homeostasis. Protein degradation, a process by which proteins are hydrolysed into amino acids, is primarily carried out by lysosomes or proteasomes. Many proteins destined for degradation are conjugated with ubiquitin (Ub) through a process known as ubiquitination, which involves the sequential action of Ub-activating enzyme (E1), Ub-conjugating enzyme (E2), and Ub-ligase (E3). The research community has harnessed this natural system to eliminate disease-causing proteins by inducing their degradation through a ubiquitin-mediated pathway. One such strategy involves PROTACs (PROteolysis TArgeting Chimeras), which are heterobifunctional molecules composed of two ligands: one binds the target protein, and the other binds an E3 Ub-ligase. This brings the E3 into proximity with the target, allowing it to catalyse the ubiquitination of the target protein, leading to its degradation.^1^

Since the advent of a heterobifunctional chimeric compound recruiting an E3 to the substrate MetAP-2 in 2001,^2^ approximately 50 substrates have been degraded with this approach,^3^ and over 25 PROTACs are currently in clinical trials.^4^ These PROTACs primarily rely on ligands that recruit either Cereblon (CRBN) or VHL E3s to promote ubiquitination and degradation of neo-substrates.^5^ However, it has been shown that CRBN- or VHL-based PROTACs do not always result in efficient ubiquitination or selective degradation of the target protein.^6,7^ Furthermore, acquired resistance to CRBN- and VHL-based PROTACs have been reported.^8–10^ Given that the human genome encodes more than 600 E3s,^11^ it is plausible that previously untapped E3s could offer distinct structural plasticity and cooperativity that favour productive ubiquitination,^12^ enabling the degradation of otherwise difficult or undruggable targets and potentially improving potency. Interest in alternative E3s for PROTAC development has increased in recent years. To date, these include DCAF1,^13^ DCAF2,^14^ DCAF11,^15^ DCAF15,^16^ DCAF16,^17^ DDB1,^18^ FBXO22,^19^ GID4,^20^ IAP,^21^ KEAP1,^22^ KLHDC2,^23^ TRIM21,^24^ MDM2,^25^ SPOP,^26^ and ZYG11B.^27^

Casitas B-cell lymphoma (CBL) is an E3 best characterised for its role in signalling pathways involving non-receptor and receptor tyrosine kinases (RTKs). Several features make CBL a promising candidate as an alternative E3 in PROTAC applications. Firstly, CBL interacts with various substrates localised to the cytosol or plasma membrane,^28^ suggesting its potential to target a broad range of neo-substrates in these compartments. Secondly, CBL mediates Ub transfer that can lead to either lysosomal or proteasomal degradation,^29–31^ which may improve degradation efficiency by utilising both degradation pathways, unlike conventional PROTACs that typically rely solely on the proteasome. Thirdly, CBL is a monomeric RING E3 that becomes catalytically active upon phosphorylation of Tyr371 in its linker helix region (LHR).^32,33^ This distinguishes it from most RING E3s used in the PROTAC system, which generally belong to the multi-subunit Cullin RING ligase family.^34–36^ In addition, the modular domain architecture of CBL enables it to ubiquitinate substrates recruited *via* its N-terminal tyrosine kinase-binding domain (TKBD), proline-rich region or C-terminal region, suggesting its catalytic RING domain has ample spatial accessibility to carry out ubiquitination. Finally, CBL is ubiquitously expressed across most human tissues (The Human Protein Atlas, http://www.proteinatlas.org/),^37^ which potentially makes CBL a pan-cancer therapeutic.

**Fig. 1:**
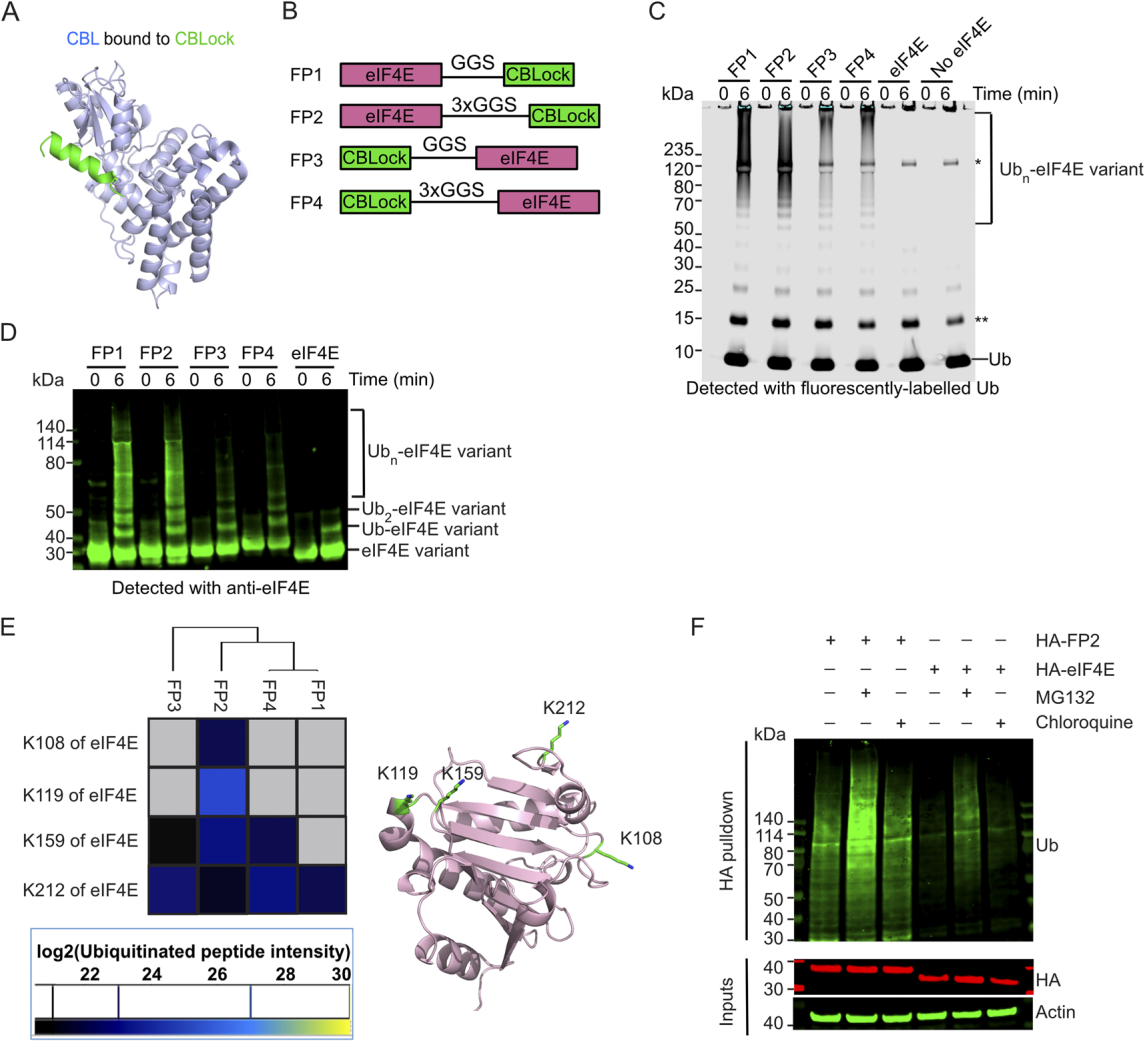
CBL ubiquitinates eIF4E-CBLock fusion protein in an *in vitro* reconstitution system and in mammalian cells. (A) Crystal structure of CBL's TKBD (light blue) bound to CBLock peptide (light green) (PDB: 9ERZ). (B) A schematic diagram showing the design of eIF4E-CBLock fusion proteins, FP1-4, where eIF4E protein (pink) is fused to either the N- or C- terminus of CBLock (green) *via* a GGS or 3xGGS linker. (C) SDS-PAGE gel showing *in vitro* ubiquitination of FP1-FP4 and controls catalysed by pTyr371-CBL detected with fluorescently-labelled Ub. A representative gel from three independent experiments is shown. The single asterisk indicates an E1-Ub band and the double asterisk indicates contamination from the fluorescently-labelled Ub. (D) Western blot showing *in vitro* ubiquitination of FP1-FP4 and controls catalysed by pTyr371-CBL detected with an anti-eIF4E antibody. A representative gel from two independent experiments is shown. (E) Mass spectrometry analysis of ubiquitination sites on eIF4E from the poly-ubiquitinated FP1-4 products (>120 kDa; SI Fig. 1B) catalysed by pTyr371-CBL. Ubiquitination sites on eIF4E are represented as a heatmap based on the abundance of peptides detected. No peptides were detected in the grey-boxed area. Ubiquitinated lysine sites on eIF4E are shown as light green sticks on a cartoon representation of the eIF4E structure (PDB: 1IPB; pink). (F) Western blots showing ubiquitination of HA-FP2 and HA-eIF4E stably expressed in HEK293 cells in the absence or presence of proteasome (MG132) or lysosomal inhibitor (chloroquine). The cell lysates were subjected to HA-tag pulldown followed by detection with an anti-Ub antibody. Cell lysates were analysed with anti-HA and anti-actin antibodies (loading control) as indicated. A representative gel from two independent experiments is shown.

Recently, we developed a peptide, CBLock, that binds selectively to the TKBD substrate-binding site of CBL with nanomolar binding affinity (Fig. 1A).^38^ In this study, we investigated whether CBL can be utilised as a degrader E3 by using CBLock as a handle to recruit neo-substrates to CBL. To demonstrate proof-of-concept, we targeted eIF4E, a key component of the eIF4F translation initiation complex, which also includes eIF4A and eIF4G, and plays a vital role in binding the 5′ cap of mRNA to facilitate translation.^39^ Overexpression of eIF4E has been linked to hallmarks of cancer such as uncontrolled cell growth and proliferation.^40–43^ Cancer-dependency analyses further show that many different cancer cell types rely heavily on eIF4E for survival (https://depmap.org/portal). Consistently, antisense oligonucleotide-mediated downregulation of eIF4E suppresses tumour growth in xenograft models.^44^ Moreover, studies in eIF4E haplo-insufficient mice revealed that a 50% reduction in eIF4E is compatible with normal development and global protein synthesis, yet is sufficient to block cellular transformation.^45^ Thus, there is an interest in targeting eIF4E for degradation by induced-proximity approaches. However, CRBN and VHL-recruiting PROTACs are so far unable to successfully degrade eIF4E in cells.^46,47^ To test if a previously unexplored CBL can be used as a degrader E3 in a PROTAC system, CBL-recruiting PROTACs, termed eIFTerminators, were generated by linking CBLock to the eIF4E-targeting sequence eIF4G1-D5S.^48^ Biochemical and cell-based analyses showed that eIFTerminators enable ternary complex formation and promote CBL-mediated eIF4E degradation as well as other components of eIF4F complex, including eIF4A and eIF4G, leading to a reduction in protein translation. These findings demonstrate the feasibility of using CBL as a degrader E3 for targeted protein degradation.

## Results and discussion

### Appending CBL-binding peptide to eIF4E facilitates its ubiquitination by CBL

To test whether CBL could serve as a degrader E3, we examined its ability to ubiquitinate a neo-substrate *in vitro* upon induced proximity. As the 16-mer peptide CBLock occupies TKBD substrate-binding site where multiple substrates are known to bind and undergo ubiquitination (Fig. 1A; Ahmed *et al.*, 2025; PDB: 9ERZ), we decided to use CBLock as a ligand to promote ubiquitination of a neo-substrate eIF4E by CBL. We fused a CBLock peptide sequence to either the N- or C-terminus of eIF4E, using short or long Gly-Gly-Ser (GGS) linkers (Fig. 1B) to facilitate CBL recruitment. The length of the GGS linkers was chosen based on a structural model of the N-terminal catalytic fragment of pTyr371-CBL bound to E2-Ub and CBLock, with eIF4E's globular body modelled near the E2-Ub active site and its N- or C-terminus proximal to CBLock's N- or C-terminus, respectively (SI Fig. 1A). These CBLock-eIF4E fusion constructs, referred to as FP1-FP4, were purified and then assessed for CBL-mediated ubiquitination.

*In vitro* ubiquitination assays were performed using recombinant UBA1 (E1), UBE2D2 (E2), fluorescent-labelled Ub and the Tyr371-phosphorylated CBL fragment (residues 47-435, pTyr371-CBL), which represents the catalytically active portion of CBL as described previously.^32^ FP1-FP4 were readily ubiquitinated by pTyr371-CBL, as indicated by the appearance of Ub ladders detected with fluorescent-Ub or Coomassie staining, which were absent in control reactions with eIF4E lacking a CBLock peptide appendage as well as reactions without any form of eIF4E (Fig. 1C and SI Fig. 1B). In addition, when detected with an anti-eIF4E antibody, FP1-FP4 showed pronounced eIF4E ladders compared to lanes containing eIF4E lacking a CBLock fusion peptide (Fig. 1D). To investigate the modification sites on FP1-FP4 and the poly-Ub chain types, we subjected the smears above the 120 kDa region, indicated in black boxes, to mass spectrometry analysis (SI Fig. 1B). Mass spectrometry confirmed that Ub was the most abundant protein identified in these smears. Various Ub linkages were detected, with K11- and K48-linked Ub peptides being the most prevalent, consistent with the promiscuous activity of UBE2D2 (SI Fig. 1C). Ubiquitination was observed at four lysine residues of eIF4E: K108, K119, K159, and K212 (Fig. 1E). These lysine sites are distributed across one face of the eIF4E protein, with K212 ubiquitination consistently detected in FP1-FP4 (Fig. 1E). Notably, FP2 was the only construct ubiquitinated at all four lysine sites. In contrast, FP1, which shares the same eIF4E-CBLock orientation as FP2 but has a shorter GGS-linker, was only ubiquitinated at K212, and the K29-Ub linkage was absent. FP4, which retains FP2's GGS-linker but has the reversed eIF4E-CBLock orientation, was only ubiquitinated at K159 and K212, with the K27-, K29-, and K33-Ub linkages being absent. These findings suggested that the spatial arrangement of eIF4E relative to CBLock influences both the ubiquitination sites on eIF4E and the resulting Ub chain patterns.

Since FP2 exhibited a greater extent of ubiquitination sites on eIF4E in a reconstituted system, we next investigated whether endogenous CBL could facilitate ubiquitination of FP2 in cells. We generated stable cell lines that constitutively express either HA-eIF4E as a control or HA-FP2. The HA-tag was appended to the N-terminus of FP2, as this sequence lacks lysine residues that could undergo ubiquitination. Previous studies have shown that EGF stimulation activates CBL E3 ligase activity *via* phosphorylation of its Tyr371.^32,49,50^ To assess the phosphorylation status of CBL Tyr371, HEK293 cells expressing Myc-tag CBL were serum-starved overnight, treated with EGF, and harvested at different time points. CBL exhibited basal Tyr371 phosphorylation, which peaked at 5 min post-stimulation and returned to baseline within 2 h (SI Fig. 1D). Based on this, we treated HA-FP2 expressing cells with EGF after serum starvation to determine whether HA-FP2 undergoes CBL-mediated ubiquitination. HA-FP2 protein levels were detectable, and treatment with the proteasome inhibitor MG132 or lysosomal inhibitor chloroquine had little impact on its abundance. To directly assess ubiquitination, we performed HA pulldown under denatured conditions followed by anti-Ub immunoblotting. HA-FP2 was poly-ubiquitinated to a greater extent than HA-eIF4E (Fig. 1F). MG132 treatment intensified the poly-ubiquitinated bands, suggesting stabilisation, whereas chloroquine had a minimal effect. These findings demonstrate that CBL can induce poly-ubiquitination of a neo-substrate and promote its degradation when brought into proximity *via* a CBL-binding peptide fusion.

### Designing peptidic PROTACs to recruit CBL for targeting eIF4E

**Fig. 2:**
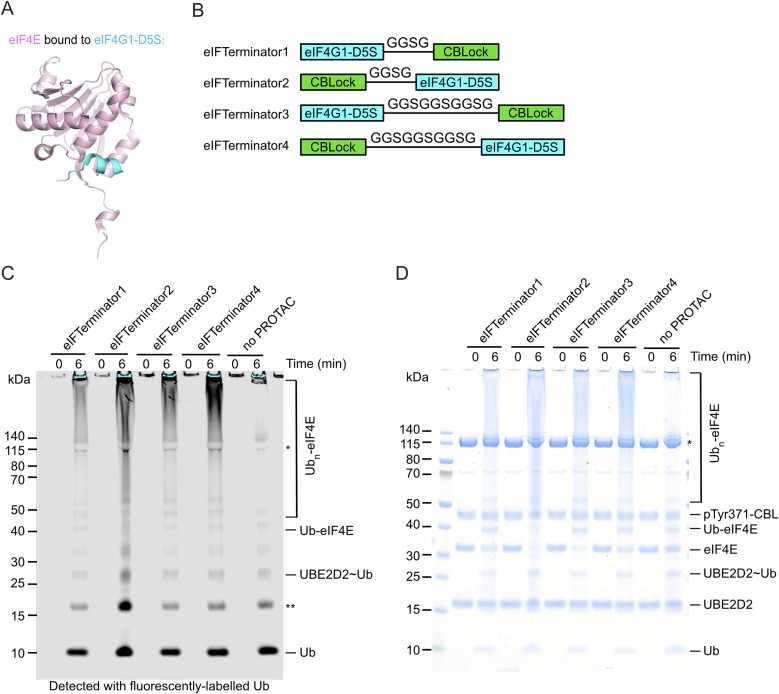
*In vitro* pTyr371-CBL-catalysed ubiquitination assay testing eIF4E ubiquitination with eIFTerminator1-4 (A) Crystal structure of eIF4E (pink) bound to eIF4G1-D5S peptide (cyan) (PDB ID: 4AZA).^48^ (B) Designs of eIF4E targeting PROTACs. The cartoons represent the peptide sequences of eIFTerminator1-4, which consist of eIF4G1-D5S and CBLock connected with a flexible GGSG or GGSGGSGGSG linker. (C) SDS-PAGE gel showing pTyr371-CBL-catalysed ubiquitination of eIF4E in the absence or presence of eIFTerminator1-4 detected with fluorescently-labelled Ub. Data are representative of three independent experiments. The single asterisk indicates the E1-Ub band and the double asterisk indicates a contaminant from the fluorescently-labelled Ub. (D) Coomassie-stained gel from C is shown. The single asterisk indicates the E1 band.

Previous studies have identified a sub-micromolar 12-mer peptide binder of eIF4E, eIF4G1-D5S, using phage display technology.^48^ The crystal structure of eIF4G1-D5S bound to eIF4E revealed that it occupies the same dorsal surface of eIF4E as the eIF4G canonical helix (Fig. 2A).^51^ To recruit CBL to eIF4E's proximity, we designed peptidic PROTACs by linking CBLock to eIF4G1-D5S. We modelled the eIF4G1-D5S-eIF4E complex onto a structural model of the pTyr371-CBL-E2-Ub-CBLock complex by simultaneously positioning eIF4G1-D5S in proximity to CBLock and eIF4E near the E2-Ub active site (SI Fig. 2A and B). The model suggested that linking eIF4G1-D5S to either terminus of CBLock would support this arrangement. Based on this, we generated four variants incorporating either a short GGSG or a long GGSGGSGGSG linker, with CBLock positioned at either terminus of eIF4G1-D5S. These constructs were named eIFTerminator1-4 (Fig. 2B).

To assess whether eIFTerminator1-4 could induce CBL-mediated eIF4E ubiquitination, we performed *in vitro* ubiquitination assays using recombinant UBA1, UBE2D2, fluorescent-labelled Ub, His-eIF4E, and pTyr371-CBL in the absence or presence of eIFTerminator1-4. All four PROTACs promoted poly-ubiquitination of eIF4E with the concomitant disappearance of the eIF4E band as compared to the control reaction lacking PROTAC (Fig. 2C and D). These findings suggest that eIFTerminator1-4 could bring CBL and eIF4E into proximity to facilitate CBL-mediated eIF4E ubiquitination.

### eIFTerminator1-4 promote the formation of the CBL-eIF4E ternary complex

To investigate the binding kinetics and affinity of eIFTerminator1-4, surface plasmon resonance (SPR) analysis was performed. SPR measures changes in the refractive index near the sensor surface, where the analyte interacts with an immobilised ligand. This allows real-time monitoring of molecular interactions, providing quantitative binding affinity and kinetic parameters. First, we assessed whether eIFTerminator1-4 designs have any impact on CBL or eIF4E binding affinity. A three-fold dilution series of the eIFTerminator1-4, starting at 1 µM, was injected over immobilised CBL to assess the protein–protein interactions between eIFTerminator1-4 to CBL (SI Fig. 3A). The binding affinities of eIFTerminator1-4 were similar (*K*_D_: 42.9–64.6 nM; Table 1 and SI Fig. 3B) and comparable to that of CBLock (*K*_D_: 122 nM; Table 1 and SI Fig. 3B). Next, we examined the interactions between eIF4E and eIFTerminator1-4 by immobilising His-eIF4E on the SPR chip and injecting a dilution series of eIFTerminator1-4. The *K*_D_ values ranged from 38.9 nM to 67.5 nM, and the binding affinity as well as kinetics were similar to that observed for the control ligand eIF4G1-D5S (Table 2 and SI Fig. 4A). These findings suggest that the design of eIFTerminator1-4 did not affect the binding affinity of the individual ligands for their respective binding partners, namely CBLock for CBL and eIF4G1-D5S for eIF4E.

**Table 1:** Binding affinities between the ligand, GST-CBL, and the analytes, eIFTerminator1-4 and CBLock. A kinetic fit to a 1 : 1 binding model was used to determine the *K*_D_ (*n* = 3, mean ± SD, except CBLock is *n* = 2, mean ± SD)

	Binary *K*_D_ (nM)
eIFTerminator1	64.6 ± 41.2
eIFTerminator2	56.9 ± 25.4
eIFTerminator3	42.9 ± 22.8
eIFTerminator4	59.9 ± 27.0
CBLock (positive ctrl)	122 ± 4.2

**Table 2:** Steady-state binding affinities between the ligand His-eIF4E and the analytes eIFTerminator1-4 or eIF4G1-D5S in a binary complex, and in a ternary complex with a fixed concentration of CBL (*n* = 3, mean ± SD)

	Binary *K*_D_ (nM)	Ternary *K*_D_: CBL (nM)
eIFTerminator1	54.0 ± 9.0	77.8 ± 9.7
eIFTerminator2	67.5 ± 7.4	60.7 ± 22
eIFTerminator3	39.5 ± 3.4	81.8 ± 8.2
eIFTerminator4	60.6 ± 8.5	43.8 ± 6.0
eIF4G1-D5S (positive ctrl)	38.9 ± 6.5	N/A

**Fig. 3:**
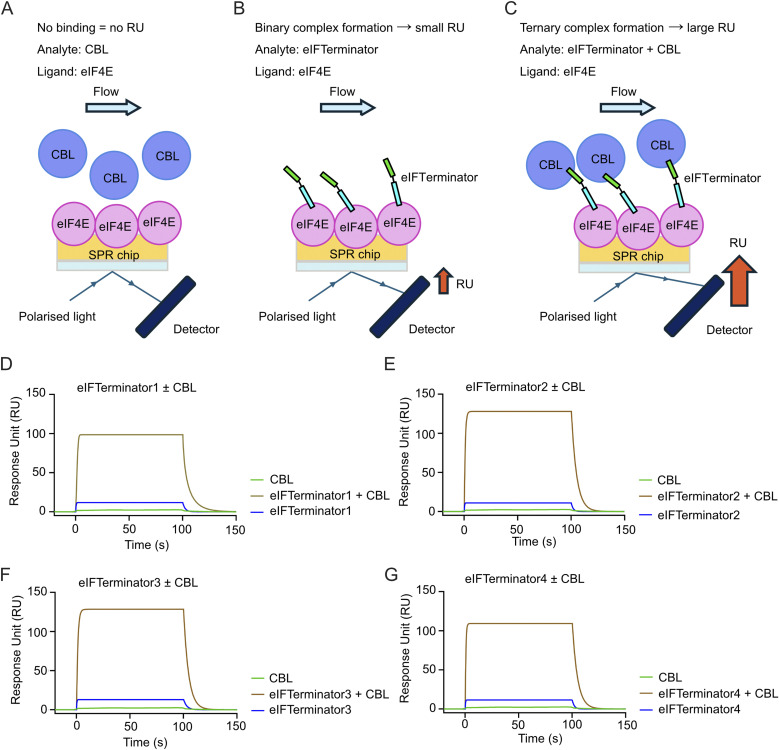
SPR showing eIFTerminator1-4 form a ternary complex with CBL and eIF4E. (A–C) Schematic illustrating the principles of ternary complex formation analysed by SPR. Briefly, eIF4E was immobilised onto the SPR sensor chip. CBL (A), eIFTerminators (B), or a mixture of eIFTerminator and a fixed concentration of CBL (C) was then flowed over the surface. Binding of an analyte to the immobilised ligand alters the refractive index near the sensor surface, generating a signal measured in response unit (RU). A small analyte, such as an eIFTerminator alone, produced a modest RU signal due to its low molecular weight. In contrast, binding of the large eIFTerminator-CBL complex generated a significantly higher RU, indicative of the ternary complex formation. (D–G) Representative sensorgrams showing changes in RU between the ligand eIF4E and the analytes eIFTerminator1-4 (D–G) alone or in the presence of CBL. eIF4E was immobilised and eIFTerminator1-4 alone (0.375 µM, blue line) or with a fixed concentration of CBL (1 µM, gold line) were injected as analytes. The line in light green represents the CBL substrate control in the absence of eIFTerminator1-4. Data are representative of three independent experiments.

In SPR, the binding of a large analyte results in a greater change in refractive index, leading to a higher response unit (RU). By immobilising eIF4E on the SPR chip, we anticipated that binding of the eIFTerminator-CBL binary complex will cause a greater change in refractive index, leading to a larger RU compared to eIFTerminator alone if the ternary complex between eIF4E, eIFTerminator and CBL has assembled (Fig. 3A–C). To investigate the ternary complex formation, a saturating concentration of CBL (1 µM), which is over five-fold higher than the *K*_D_ for the CBL-CBLock interaction, was included with the eIFTerminator1-4 analytes and flowed over immobilised eIF4E. Addition of CBL alone, in the absence of eIFTerminators, caused little change in RU, indicating that CBL did not interact with eIF4E (Fig. 3D–G). When eIFTerminator1-4 was injected alone, there was an increase of 10–15 RU. In contrast, in the presence of CBL, there was an increase of 100-120 RU (Fig. 3D–G), confirming ternary complex formation. In addition, since positive cooperativity in ternary complexes has been shown to influence degradation efficiency,^12^ we compared the eIFTeminator1-4 binding affinities for eIF4E in binary complexes to ternary complexes in the presence of 1 µM CBL. The *K*_D_ values and binding kinetics were similar (Table 2 and SI Fig. 4B), suggesting no cooperative effect. Together, these findings demonstrate that eIFTerminators bind both CBL and eIF4E to form the ternary complex.

### CP-eIFTerminator4 reduces the level of eIF4E in a dose-dependent manner

To examine whether eIFTerminator1-4 could induce eIF4E degradation in cells, we incorporated a 16-mer penetratin peptide sequence from the *Drosophila* Antennapedia homeodomain^52^ at their N-terminus to facilitate cell entry. Additionally, an N-terminal FITC label was included to monitor cell uptake. These modified peptidic PROTACs are referred to as cell-penetrating eIFTerminator1-4 (CP-eIFTerminator1-4). Among them, CP-eIFTerminator1, CP-eIFTerminator3, and CP-eIFTerminator4 readily dissolved in water or DMSO, whereas CP-eIFTerminator2 was insoluble and therefore not pursued further.

A key consideration when characterising PROTACs in cells is cytotoxicity, as compounds that significantly reduce cell viability within a few hours may exert off-target cytotoxic effects rather than on-target degradation. To assess this, cell viability assays were performed after incubating HeLa cells with CP-eIFTerminators at concentrations ranging from 0.16–24.4 µM for 3 h and 8 h (SI Fig. 5A and B). The parental cell-penetrating peptides CP-CBLock and CP-eIF4G1-D5S were included as controls. The assay showed that CP-eIF4G1-D5S exhibited cytotoxicity at concentrations of 16.10 µM or higher, reducing viable cells to as low as 35% at 3 h and 25% at 8 h post-peptide treatment, whereas lower concentrations (0.16–16.10 µM) had no apparent effect on cell viability (SI Fig. 5A and B). In contrast, CP-CBLock and CP-eIFTerminator4 did not affect cell viability under the tested conditions. However, CP-eIFTerminator1 and CP-eIFTerminator3 showed mild cytotoxic effects at higher concentrations (16.10–24.4 µM). Although it remains unclear why the eIF4E warhead eIF4G1-D5S exhibited cytotoxicity at higher concentrations, it is intriguing that linking CBLock to its N-terminus in CP-eIFTerminator4 mitigates the cytotoxicity.

**Fig. 4:**
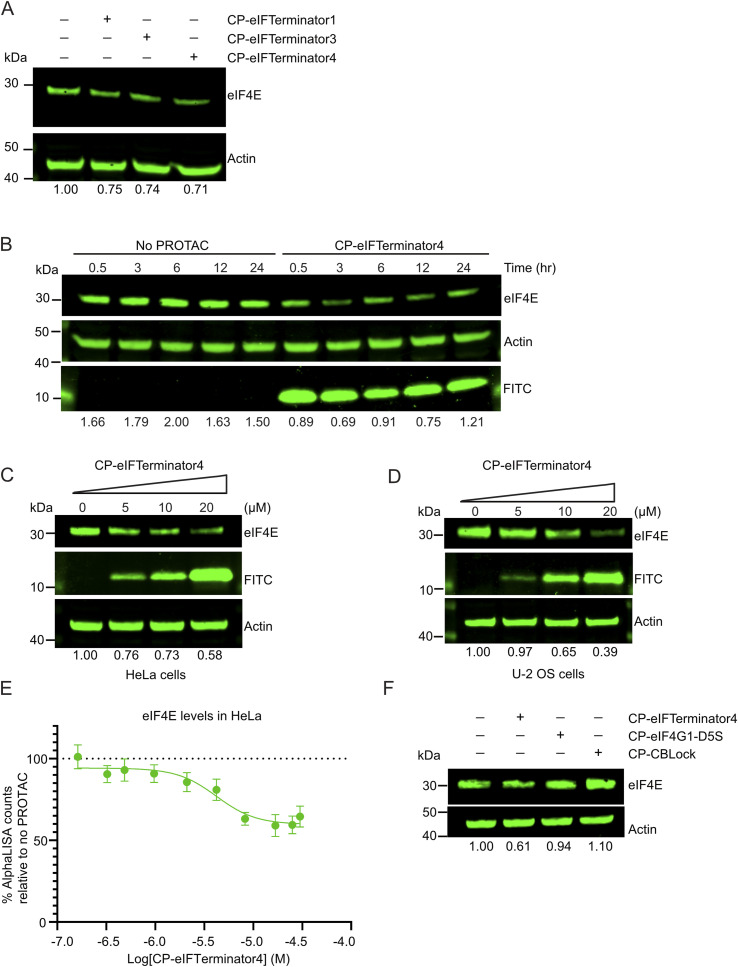
CP-eIFTerminators facilitate eIF4E degradation in cells. (A) Western blots showing eIF4E and actin (loading control) levels from lysates of HeLa cells treated with water (no PROTAC), 10 µM CP-eIFTerminator1, CP-eIFTerminator3, and CP-eIFTerminator4 for 3 h. The band intensities of eIF4E from each lane were quantified and normalised using actin band intensities and shown as a ratio relative to the no PROTAC control lane. Data are representative of three independent experiments. (B) Western blots showing the loading control actin, eIF4E and FITC levels from lysates of HeLa cells treated with 20 µM FITC-labelled CP-eIFTerminator4 or water (no PROTAC) over indicated times. The band intensities of eIF4E from each lane were quantified and normalised using actin band intensities. Data are representative of two independent experiments. (C) Western blots showing actin (loading control), eIF4E, and FITC levels from lysates of HeLa cells treated with 5, 10, and 20 µM of CP-eIFTerminator4 for 3 h. Water was added as a negative control, indicated as 0 µM. The band intensities of eIF4E from each lane were quantified and normalised using actin band intensities and shown as a ratio relative to the no PROTAC control lane. Data are representative of two independent experiments. (D) Western blots showing the loading control actin, eIF4E, and FITC levels from lysates of U-2 OS cells treated with 5, 10, and 20 µM of FITC-labelled CP-eIFTerminator4 for 3 h. Water was added as a negative control, indicated as 0 µM. The band intensities of eIF4E from each lane were quantified and normalised using actin band intensities and shown as a ratio relative to the no PROTAC control lane. Data are representative of two independent experiments. (E) An AlphaLISA assay showing a dose–response curve when HeLa cells are treated with CP-eIFTerminator4 for 3 h eIF4E in HeLa cells was detected using acceptor and donor beads for quantification. The datapoint associated with the hook effect was omitted when calculating DC_50_ (*n* = 3 repeated experiments, mean ± SEM). (F) Western blots showing actin (loading control) and eIF4E levels from lysates of HeLa cells treated with water (no PROTAC), 10 µM CP-eIFTerminator4, CP-eIF4G1-D5S, or CP-CBLock for 3 h. The band intensities of eIF4E from each lane were quantified and normalised using actin band intensities and shown as a ratio relative to the no PROTAC control lane. Data are representative of three independent experiments.

Based on these findings, we treated HeLa cells with 10 µM of CP-eIFTerminator1, CP-eIFTerminator3 or CP-eIFTerminator4 and assessed the effects on the endogenous levels of eIF4E. HeLa cells were chosen, due to their high *EIF4E* and *CBL* mRNA expression (The Human Protein Atlas, https://www.proteinatlas.org/). Treatment with CP-eIFTerminator1, CP-eIFTerminator3 or CP-eIFTerminator4 resulted in a similar extent of reduction in eIF4E protein levels, namely a reduction between 24.6% and 28.7%, compared to the no PROTAC treatment control (Fig. 4A). CP-eIFTerminator4 was selected for further testing, as it did not cause acute cytotoxicity at 3-h and 8-h post treatment (SI Fig. 5A and B), which enabled the assessment of its potency at higher concentrations.

Next, a time course experiment was performed to assess whether the reduction in eIF4E levels was sustained over time. HeLa cells were incubated with 20 µM CP-eIFTerminator4 and examined at selected time points over a 24-h period. Western blot analysis showed minimal changes in FITC levels over the 24-h period (Fig. 4B). CP-eIFTerminator4 readily entered cells as soon as 30 min and caused a reduction in eIF4E protein levels between 0.5–12 h. Although eIF4E levels gradually increased by 24 h, the level was still lower in CP-eIFTerminator4-treated cells compared to untreated cells. Since levels of eIF4E were most reduced at 3 h following CP-eIFTerminator4 treatment, we opted to further characterise this PROTAC at the 3-h time point.

Two cancer cell lines, HeLa and U-2 OS, were chosen based on their high mRNA expression of *CBL* and *EIF4E* (The Human Protein Atlas, https://www.proteinatlas.org/), in order to validate that CP-eIFTerminator4 exerts its effect in a concentration-dependent manner. HeLa cells were treated with varying concentrations of CP-eIFTerminator4 and harvested after 3 h for immunoblotting analysis. A concentration-dependent decrease in eIF4E levels was observed (Fig. 4C). Similarly, U-2 OS cells treated under comparable conditions showed a reduction in intracellular eIF4E levels as the concentration of CP-eIFTerminator4 was increased (Fig. 4D). Since the two cancer cell lines exhibited similar results, we selected HeLa cells for further characterisation.

Next, we examined the degradation efficiency of CP-eIFTerminator4 in HeLa cells using AlphaLISA. Cells were treated with a titration series of CP-eIFTerminator4 (0.49–30.07 µM) for 3 h, followed by AlphaLISA analysis to quantify intracellular eIF4E levels. CP-eIFTerminator4 showed a half-maximal degradation concentration (DC_50_) of approximately 4.66 µM and a maximum degradation (*D*_max_) of 39.4% (Fig. 4E). Since it was not possible to prepare eIF4E-knockout HeLa cells as a negative control, background subtraction was done using empty wells instead. For this reason, it was unclear if the true extent of degradation was under-represented by the measured *D*_max_. In comparison, quantification of eIF4E bands on immunoblots from four independent samples treated with 20 µM CP-eIFTerminator4 for 3 h yielded an average *D*_max_ of 44.0% (SD ± 18.6%). The AlphaLISA and western blot data were similar, and the extent of degradation varied more across experiments in the latter case. Nonetheless, the lack of an eIF4E knock-out negative control is unlikely to significantly affect the determination of the DC_50_.

To determine whether the degradation of eIF4E by CP-eIFTerminator4 is driven by an induced-proximity mechanism rather than by the individual warheads, we tested CP-eIFTerminator4, CP-eIF4G1-D5S, and CP-CBLock separately in HeLa cells (Fig. 4F). Since CP-eIF4G1-D5S affected cell viability at concentrations above 16 µM while CP-CBLock and CP-eIFTerminator4 did not affect cell viability (SI Fig. 5A and B), all compounds were tested at 10 µM. Under this condition, only CP-eIFTerminator4 significantly reduced eIF4E (Fig. 4F). This confirms that CP-eIFTerminator4 functions as a PROTAC and that the individual warheads do not trigger eIF4E degradation.

Degradation of eIF4E by an artificial induced-proximity approach using a dTAG system has been shown to be feasible.^53^ However, degradation of endogenous eIF4E *via* a PROTAC approach using CRBN and VHL ligands has remained challenging; despite achieving cell entry and ternary complex formation, these PROTACs failed to induce eIF4E degradation.^46,47^ Our work presents the first case in which eIF4E is targeted for degradation in a PROTAC system.

### CP-eIFTerminator4 recruits CBL and induces eIF4E degradation using two cellular degradation pathways

**Fig. 5:**
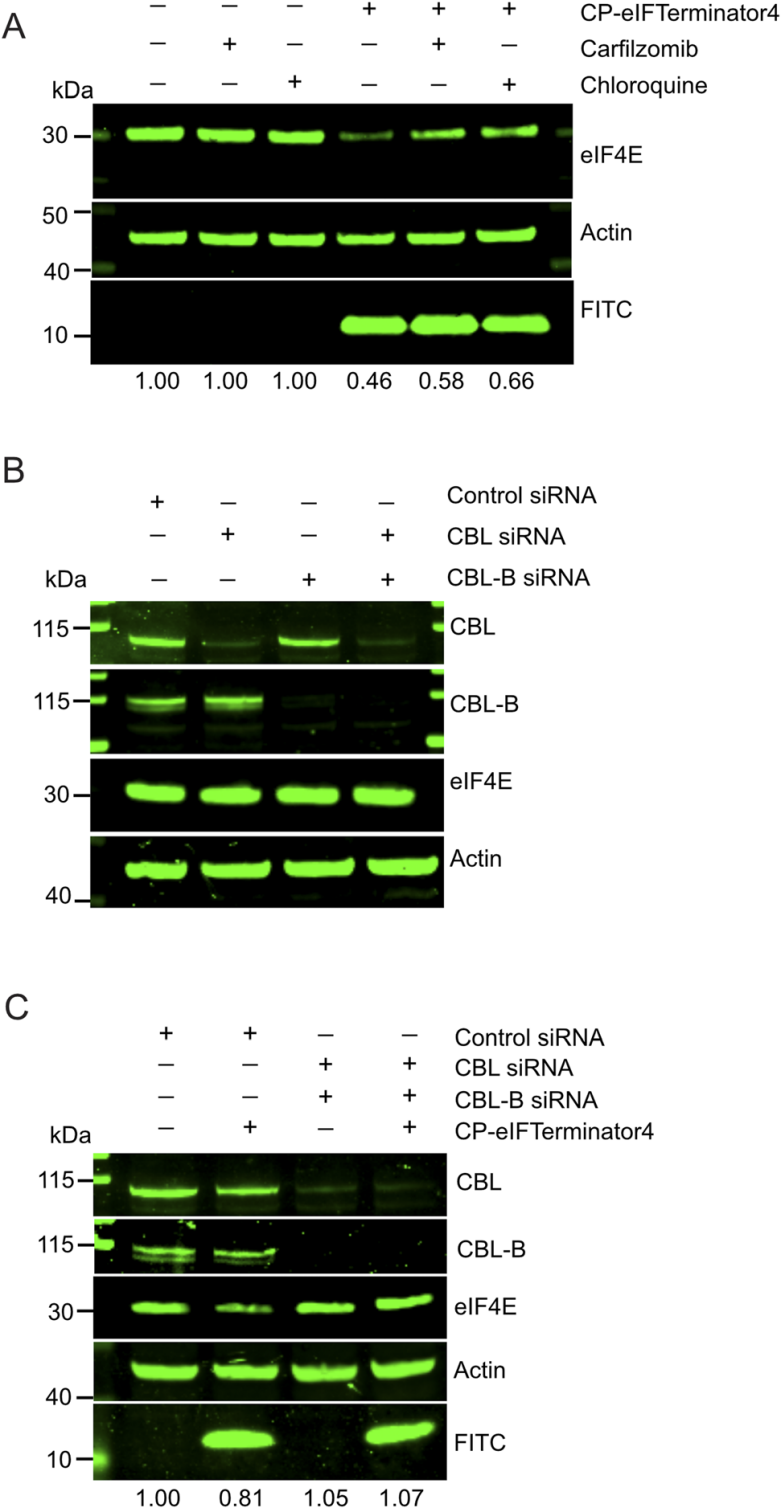
CP-eIFTerminator4 promotes CBL proteins-mediated eIF4E degradation *via* lysosomal and proteasomal pathways. (A) Western blots showing the loading control actin, eIF4E, and FITC from HeLa cell lysates treated with FITC-CP-eIFTerminator4 for 3 h. Cells were pre-treated with carfilzomib or chloroquine for 4 h prior to the PROTAC treatment to block either the proteasomal or lysosomal pathways. The band intensities of eIF4E from each lane were quantified and normalised using actin band intensities and shown as a ratio relative to the no PROTAC control lane. Data are representative of three independent experiments. (B) Western blot demonstrating knockdown of CBL and/or CBL-B after 72 h siRNA treatment in HeLa cells. Cell lysates were probed with antibodies against CBL, CBL-B, eIF4E, and anti-actin (integrity control). Data are representative of two independent experiments. (C) Western blot showing eIF4E rescue in HeLa cells co-treated with CBL and CBL-B siRNA in the presence of CP-eIFTerminator4. Cells were treated with siRNA for 72 h prior to 20 µM CP-eIFTerminator4 treatment for 3 h. Lysates were probed for the loading control actin, CBL, CBL-B, eIF4E, and FITC. The band intensities of eIF4E from each lane were quantified and normalised using actin band intensities and shown as a ratio relative to the no PROTAC control lane (*n* = 2).

Since degradation was observed in the initial hours following CP-eIFTerminator4 treatment (Fig. 4B), the next step was to investigate the mechanism of degradation. To determine which degradation pathway is responsible for eIF4E reduction, HeLa cells were treated with 20 µM CP-eIFTerminator4 alongside the proteasome inhibitor carfilzomib or the lysosomal inhibitor chloroquine. Carfilzomib, an irreversible proteasomal inhibitor, was pre-incubated at a concentration of 100 nM, while chloroquine was used at a concentration of 50 µM for 4 h prior to adding CP-eIFTerminator4 for 3 h. Inhibition of protein degradation *via* either proteasome or lysosome led to eIF4E stabilisation (Fig. 5A).

We previously showed that CBLock binds to both CBL and CBL-B, as both CBL proteins share identical sequences at the TKBD substrate-binding site targeted by CBLock.^38^ To determine whether eIF4E degradation by CP-eIFTerminator4 is mediated by the CBL proteins, we performed siRNA knockdown of *CBL* and *CBL-B* in HeLa cells. We confirmed that *CBL* siRNA and *CBLB* siRNA were able to deplete CBL or CBL-B, respectively, in HeLa cells (Fig. 5B). In addition, knockdown of CBL and/or CBL-B had no effect on eIF4E protein levels. As expected from a peptide that recruits CBL, co-treatment of *CBL* and *CBLB* siRNA inhibited CP-eIFTerminator4-mediated eIF4E degradation (Fig. 5C). These findings demonstrate that CBL proteins are required for CP-eIFTerminator4-mediated degradation of eIF4E and suggest that CBL and CBL-B can facilitate eIF4E degradation through both lysosomal and proteasomal pathways.

Unlike lysosome-targeting chimeras (LYTACs), which rely on lysosomal targeting receptors such as the cation-independent mannose-6-phosphate receptor or the asialoglycoprotein receptor,^54^ or autophagy-targeting chimeras (AUTACs), which induces autophagosome formation through *S*-guanylation,^55^ our study, to our knowledge, represents the first example of a PROTAC hijacking a cytoplasmic E3 to degrade a cytoplasmic neo-substrate *via* the lysosomal pathway. It remains unclear which types of Ub chains are assembled on the neo-substrate by CBL in cells to direct it toward either lysosomal or proteasomal degradation. However, the ability to engage both degradation pathways may offer a distinct advantage by increasing the likelihood of effective neo-substrate clearance. Interestingly, a recent study showed that the natural CBL substrate Lyn kinase was degraded by both CBL and CBL-B upon treatment with the Lyn-targeting inhibitor SI-3 (Scholes, Bertoni^56^). This degradation involved both lysosomal and proteasomal pathways. Together, these results suggest that CBL proteins can mediate substrate degradation through distinct mechanisms depending on cellular context and pharmacological cues.

### CP-eIFTerminator4 disrupts the eIF4F complex and translation

**Fig. 6:**
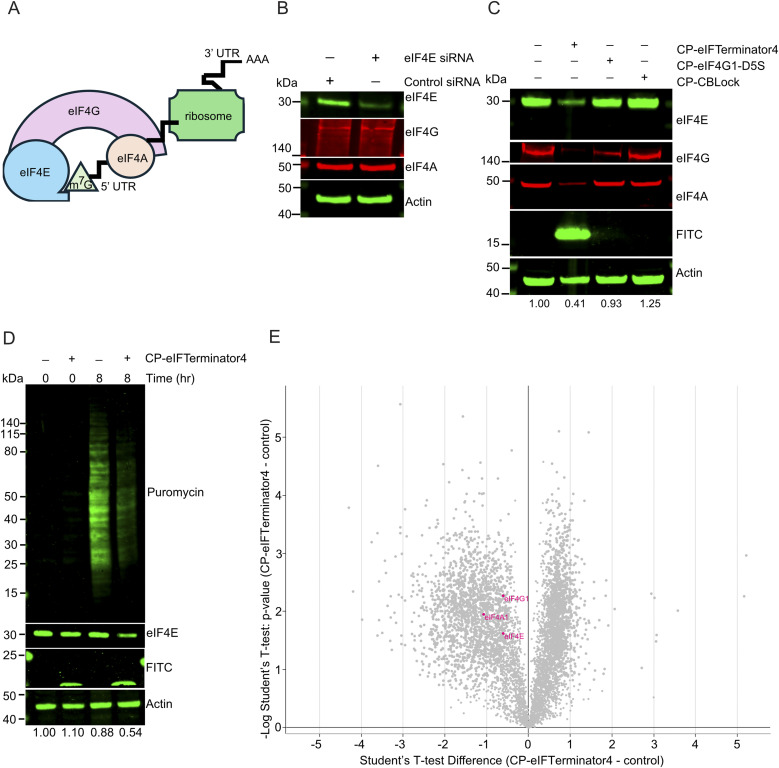
CP-eIFTerminator4 has an on-target effect on eIF4F-dependent translation through degradation of eIF4E. (A) A schematic illustration of the eIF4F complex consisting of eIF4A, eIF4E, and eIF4G in cap-dependent translation. eIF4E is critical for cap-dependent translation as it binds to the 5′ cap of mRNA that consists of 7-methylguanosine (m^7^G). eIF4G serves as a scaffold for the RNA helicase eIF4A. (B) Western blot showing the levels of eIF4A, eIF4E, eIF4G, and actin (loading control) from cell lysates of HeLa cells treated with *EIF4E* siRNA or control siRNA for 96 h. Data are representative of three independent experiments. (C) Western blot showing the levels of eIF4E, eIF4G, eIF4A, FITC, and actin (loading control) from lysates of HeLa cells treated with CP-eIFTerminator4, CP-eIF4G1-D5S or CP-CBLock for 3 h. The band intensities of eIF4E from each lane were quantified and normalised using actin band intensities and shown as a ratio relative to the no PROTAC control lane. Data are representative of three independent experiments. (D) Western blots showing a puromycin incorporation assay demonstrating reduction in translation when HeLa cells are treated with 20 µM CP-eIFTerminator4 for 8 h. Puromycin was added 1 h prior to harvest for the 8-h time point, while cells were harvested immediately after puromycin was added to the cell culture for 0-h time point samples. Anti-actin was used as an integrity control, anti-eIF4E was used to validate eIF4E degradation, anti-FITC was used to confirm CP-eIFTerminator4 cell entry, and an anti-puromycin antibody was used to visualise the newly synthesised peptides. Data are representative of three independent experiments. (E) Volcano plot showing differences in the proteome of HeLa cells treated with CP-eIFTerminator4 compared to untreated control. The experiment was performed in three biological replicates. Each dot represents a protein whose abundance increased (right side) or decreased (left side) upon CP-eIFTerminator4 treatment. Dots in pink highlight the components of the eIF4F complex.

Since the eukaryotic cap-dependent translation complex eIF4F consists of the RNA helicase eIF4A1, mRNA cap-binder eIF4E, and the scaffold protein eIF4G1 (Fig. 6A), we sought to assess whether the depletion of eIF4E also affects the protein levels of eIF4A1 and the scaffold eIF4G1 concomitantly. Consistent with previous reports,^45,57^*EIF4E* siRNA treatment in HeLa cells for 96 h did not perturb eIF4A1 or eIF4G1 levels (Fig. 6B). In contrast, 3 h of CP-eIFTerminator4 treatment in HeLa cells led to a reduction in eIF4A1, eIF4E, and eIF4G1 levels (Fig. 6C). The warheads, CP-eIF4G1-D5S or CP-CBLock, had minimal effects on eIF4A, eIF4E, and eIF4G levels (Fig. 6C). Combined, it appears that CP-eIFTerminator4 acts on the eIF4F complex *via* a unique mechanism that is distinct from siRNA-mediated knockdown of eIF4E, or occupancy-driven inhibition mediated by eIF4G1-D5S.

To gain insight into the effect on translation as a result of depletion of the core eIF4F components, HeLa cells were pre-treated with or without CP-eIFTerminator4 for 7 h, followed by addition of puromycin for 1 h (Fig. 6D). Puromycin is a tyrosyl-tRNA mimetic that inhibits translation, but it is widely used to monitor the rate of protein synthesis.^58,59^ It binds to the ribosome during translation and is incorporated into the nascent polypeptide chain, causing premature termination and the release of puromycin-labelled peptides, which can be detected using an anti-puromycin antibody. Treatment with CP-eIFTerminator4 for 8 h was found to reduce polypeptide formation by 61.5% (SD ± 23.2, *n* = 3) (Fig. 6D).

As the puromycin incorporation assay suggested that translation is impaired in HeLa cells treated with CP-eIFTerminator4, we next performed a quantitative expression proteomics experiment to examine changes in the global proteome. Three independent replicates of HeLa cell treated with CP-eIFTerminator4 for 3 h were compared to untreated controls. Consistent with the puromycin incorporation data (Fig. 6D), CP-eIFTerminator4 treatment induced a widespread change in the proteome (Fig. 6E). The asymmetric volcano plot highlights a stronger trend towards downregulation, and many proteins were reduced to a greater extent than eIF4E. Proteomic analysis revealed that 1207 proteins were upregulated and 1779 were downregulated (SI Data 1). Notably, eIF4A1, eIF4E, and eIF4G1 showed a statistically significant reduction as a result of CP-eIFTerminator4 treatment. These findings suggest that perturbation of eIF4E and the eIF4F complex results in extensive downstream proteome remodelling.

Further KEGG and Reactome pathway analyses of the proteomics data revealed that enzymes involved in tRNA aminoacylation, the pentose phosphate pathway, and fructose and mannose metabolism were downregulated. In particular, 11 out of the 12 detected aminoacyl-tRNA synthetases were decreased, raising the possibility that their reduced levels may further compromise protein synthesis (SI Data 1).

Given the essential role of eIF4E in cap-dependent translation, our proteomics data do not allow a clear distinction between direct and indirect effects of CP-eIFTerminator4 on the proteome. It also remains to be determined how eIF4A and eIF4G are downregulated by CP-eIFTerminator4 treatment. Nevertheless, in light of previous studies such as the 30% reduction in [^35^S]methionine incorporation into the newly synthesised proteins despite an 80–90% decrease in eIF4E levels in rabbit reticulocytes,^60^ and the largely unchanged global translation in eIF4E haplo-sufficient mice,^45^ our results demonstrate that targeted degradation of eIF4E and other components of eIF4F complex by CP-eIFTerminator4 produces a substantial impact on translation.

## Conclusion

Targeted protein degradation (TPD) *via* PROTACs has expanded rapidly over the past decade. While an increasing number of E3s have been exploited for PROTAC application, there remains a strong demand to diversify the E3 toolbox, particularly to address undruggable substrates, tissue-specific E3 expression limitations, and emerging drug resistance.

In this study, we investigated the potential of the previously uncharacterised E3, CBL, as a degrader E3 to target neo-substrates for ubiquitination and degradation. We designed peptidic PROTACs, eIFTerminators, by linking CBLock to an eIF4E-binding peptide, eIF4G-D5S. We demonstrated that eIFTerminators promote ternary complex formation between CBL, eIFTerminator, and eIF4E, resulting in CBL-mediated eIF4E ubiquitination *in vitro*. In cells, eIFTerminator4 rapidly targets eIF4E for degradation *via* both lysosomal and proteasomal pathways in a manner dependent on CBL and CBL-B. Unexpectedly, eIFTerminator4 also led to the depletion of eIF4A and eIF4G, resulting in a marked reduction in global protein translation. Using a range of biochemical, biophysical, and cell-based assays, we have established CBL as a viable degrader E3 for TPD applications.

Unlike siRNA, which typically requires 24–72 h to show results, or CRISPR-Cas9, which involves months of intensive effort to knock out a gene, CP-eIFTerminator4 achieved intracellular eIF4E degradation in 30 min simply by adding the peptide to cell culture media with EGF. Furthermore, CP-eIFTermiantor4 did not acutely affect cell viability. Thus, our eIF4E PROTACs may be a useful chemical tool to study essential survival genes with minimal disruption of cellular systems.

Importantly, our CBL PROTAC was able to degrade an oncoprotein that is regarded undruggable with other E3s or inhibitors,^46,47,53,61,62^ highlighting its potential to expand the scope of PROTAC-based anti-cancer therapies. Future studies are required to elucidate the precise mechanism of action of CP-eIFTerminator4. Nonetheless, designing a PROTAC that targets the entire eIF4F complex for degradation may represent an interesting avenue for cancer therapy. Furthermore, a small molecule targeting the CBL's substrate-binding site, similar to eIFTerminator4, has recently been reported.^63^ In contrast to CBL ligands that bind the LHR site and inhibit CBL E3 activity,^64,65^ this compound does not interfere with CBL's E3 activity. Combined with previously described small-molecule inhibitors of eIF4E,^53,62,66–68^ it could enable the development of next-generation CBL-based eIF4E PROTACs with improved potency.

## Author contributions

A. T. W, A. H., and D. T. H. conceived the project. A. T. W and D. T. H. wrote the manuscript. A. T. W, L. B, T. S. generated constructs and purified proteins. A. T. W. performed all experiments under the guidance and scientific input of L. B., T. S., T. S., A. M.-J., J. T, A. G, M. B, A. K. H. and D. T. H. S. L. performed and analysed mass spectrometry data. A. T. W., J. T., and A. G. analysed SPR data. All authors commented on the manuscript.

## Conflicts of interest

A. T. W., L. B., T. S., T. S., S. L., M. B., D. T. H. are current employees of the Cancer Research UK Scotland Institute. The above authors declare no competing interests. A. M.-J., J. T, A. G., and A. K. H. are employees of AstraZeneca and have stock ownership and/or stock options and commercial interests in the company.

## Supplementary Material

SC-017-D5SC06141E-s001

SC-017-D5SC06141E-s002

## Data Availability

Raw mass spectrometry proteomics data have been deposited to the ProteomeXchange Consortium (http://proteomecentral.proteomexchange.org) *via* the PRIDE partner repository with the dataset identifiers PRIDE: PXD063494 and PXD071639. All data generated during this study are included in this article or are available from the corresponding author upon request. All raw gels, western blot images, AlphaLISA data, and SPR data generated during this study have been deposited to Mendeley (https://data.mendeley.com/preview/ptcdwrhj2x?a=b14d0213-7a88-4820-bd0c-2506dfc23dcc). Supplementary information (SI) is available. See DOI: https://doi.org/10.1039/d5sc06141e.
