# Supplementary material for: CBL ubiquitin ligase targets translation as a degrader E3
Source: Chem Sci. 2025 Dec 26;17(8):4061–74. doi: 10.1039/d5sc06141e (PMC12774438; doi:10.1039/d5sc06141e)
Supplement: SC-017-D5SC06141E-s002 [file SC-017-D5SC06141E-s002.pdf]

Supplementary Information (SI) for Chemical Science.
This journal is © The Royal Society of Chemistry 2025

Supplementary information

CBL ubiquitin ligase targets translation as a degrader E3

Alice T. Wicks, Lori Buetow, Toshiyasu Suzuki, Tobias Schmidt, Sergio Lilla, Abigail Macmillan-Jones, Jennifer Turney, Andrea Gohlke, Martin Bushell, Andreas K. Hock, Danny T. Huang

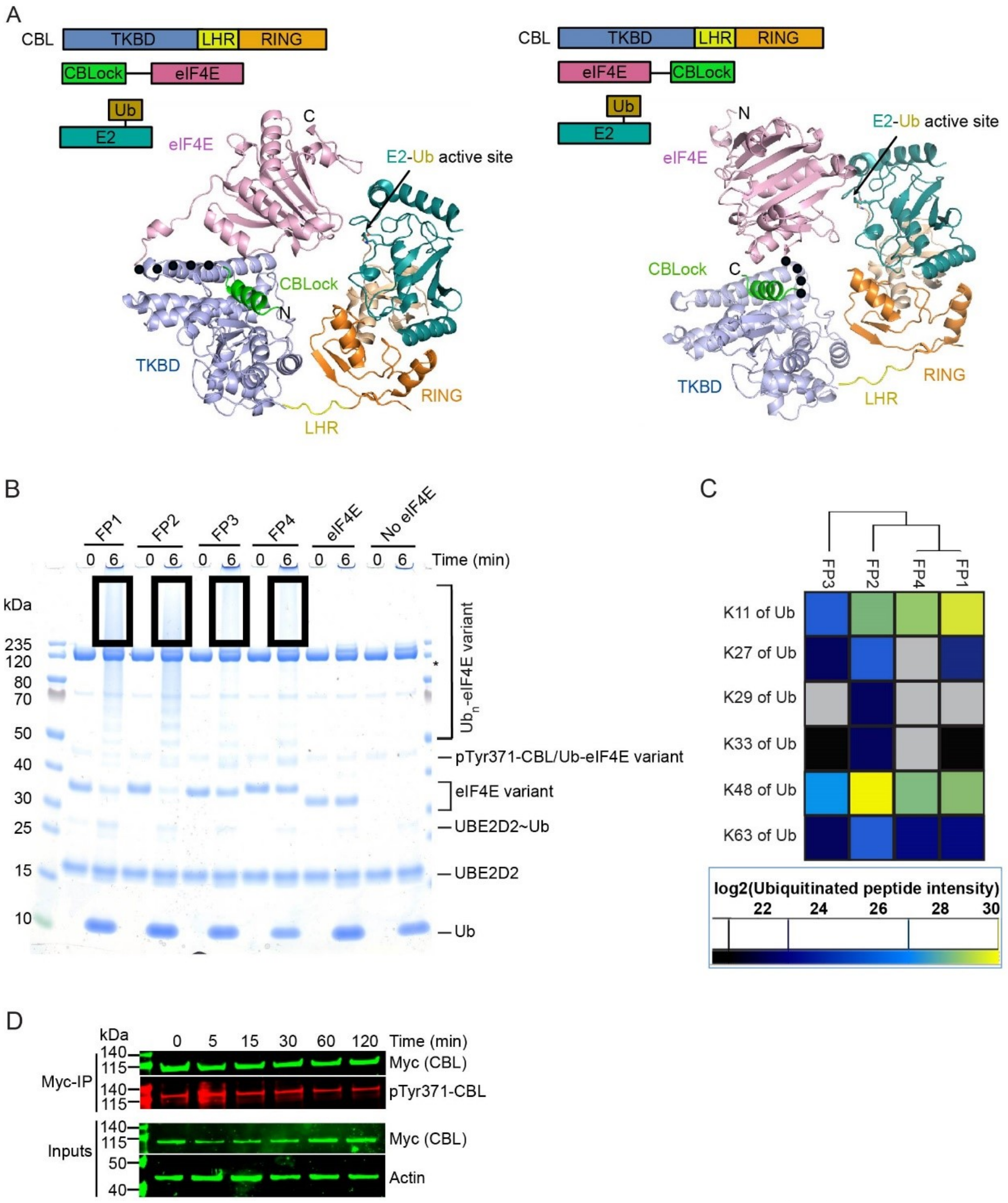

Supplementary Figure 1. Related to **Figure 1**

A. Structural models of CBL bound to E2-Ub and CBLock with eIF4E positioned close to the E2-Ub active site. The structure of CBL containing the N-terminal TKBD-LHR-RING domain bound to E2 (PDB: 4A4C; TKBD in light blue, LHR in yellow, RING in orange and E2 in dark teal) was modelled with E2-Ub (from PDB: 3ZNI) and CBLock (from PDB: 9ERZ). The structure of eIF4E (PDB: 1IPB (1); pink) was then modelled near the E2-Ub active site with eIF4E's N-terminus positioned near the C-terminus of CBLock (left panel) or with eIF4E's C-terminus positioned near the N-

terminus of CBLock (right panel). Black dots indicate the linker connecting eIF4E and CBLock.

B. Coomassie-stained SDS-PAGE gel from **Figure 1C**. The black boxes indicate the areas that were subsequently analysed by mass spectrometry to detect the ubiquitination site on eIF4E and Ub linkages. The asterisk indicates the E1 band.

C. Mass spectrometry analysis of ubiquitination sites on Ub from the poly-ubiquitinated FP1-4 products (>120 kDa; **Supplementary Figure 1B**) catalysed by pTyr371-CBL. Ubiquitination sites on Ub are represented as a heatmap based on the abundance of peptides detected. The grey boxes indicate no peptide was detected.

D. Western blots showing the levels of CBL Tyr371 phosphorylation after EGF stimulation. Myc-CBL was overexpressed in HEK293 cells for 48 hr. Subsequently, EGF was introduced, and cells were harvested. Myc-IP was performed and an anti-Myc antibody and an anti-pTyr371-CBL antibody were used for detection. An anti-actin antibody was used to probe for actin as an integrity control.

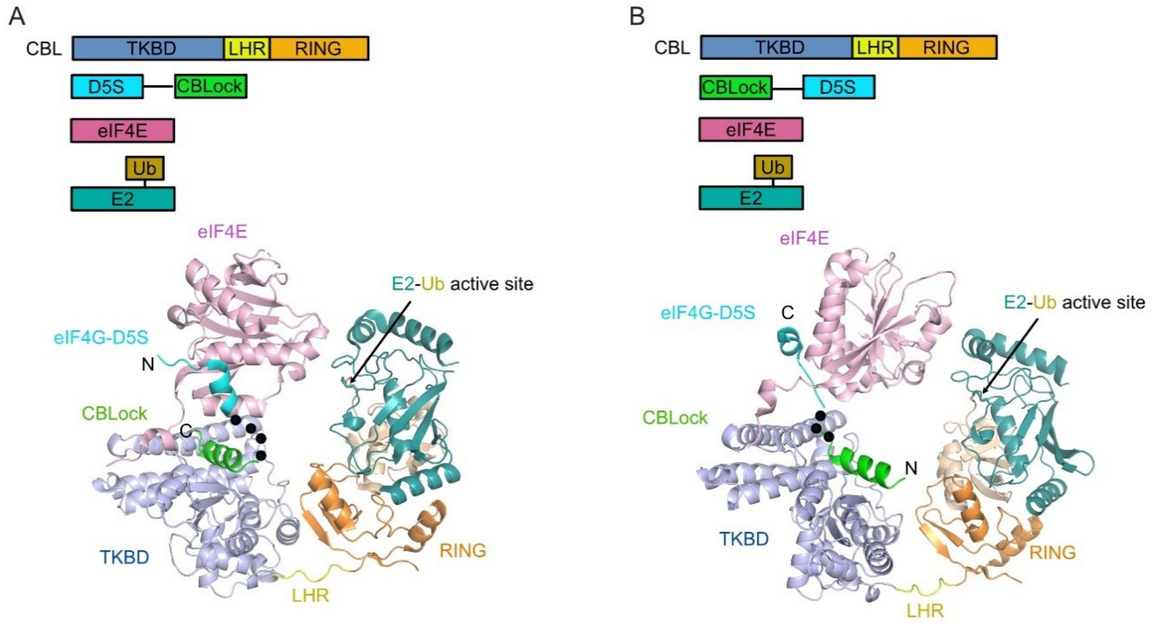

Supplementary Figure 2. Related to Figure 2

Structural models of CBL bound to E2-Ub and CBLock with eIF4E-eIF4G1-D5S complex positioned close to the E2-Ub active site and CBLock. The structure of CBL containing the N-terminal TKBD-LHR-RING domain bound to E2 (PDB: 4A4C; TKBD in light blue, LHR in yellow, RING in orange and E2 in dark teal) was modelled with E2-Ub (from PDB: 3ZNI) and CBLock (from PDB: 9ERZ). The structure of the eIF4E-eIF4G1-D5S complex (PDB: 4AZA; eIF4E in pink and eIF4G1-D5S in cyan) was then modelled with eIF4E's globular body facing towards the E2-Ub active site and eIF4G1-D5S positioned near the N-terminus (A) or the C-terminus (B) of CBLock. Black dots indicate the linker connecting eIF4G1-D5S and CBLock.

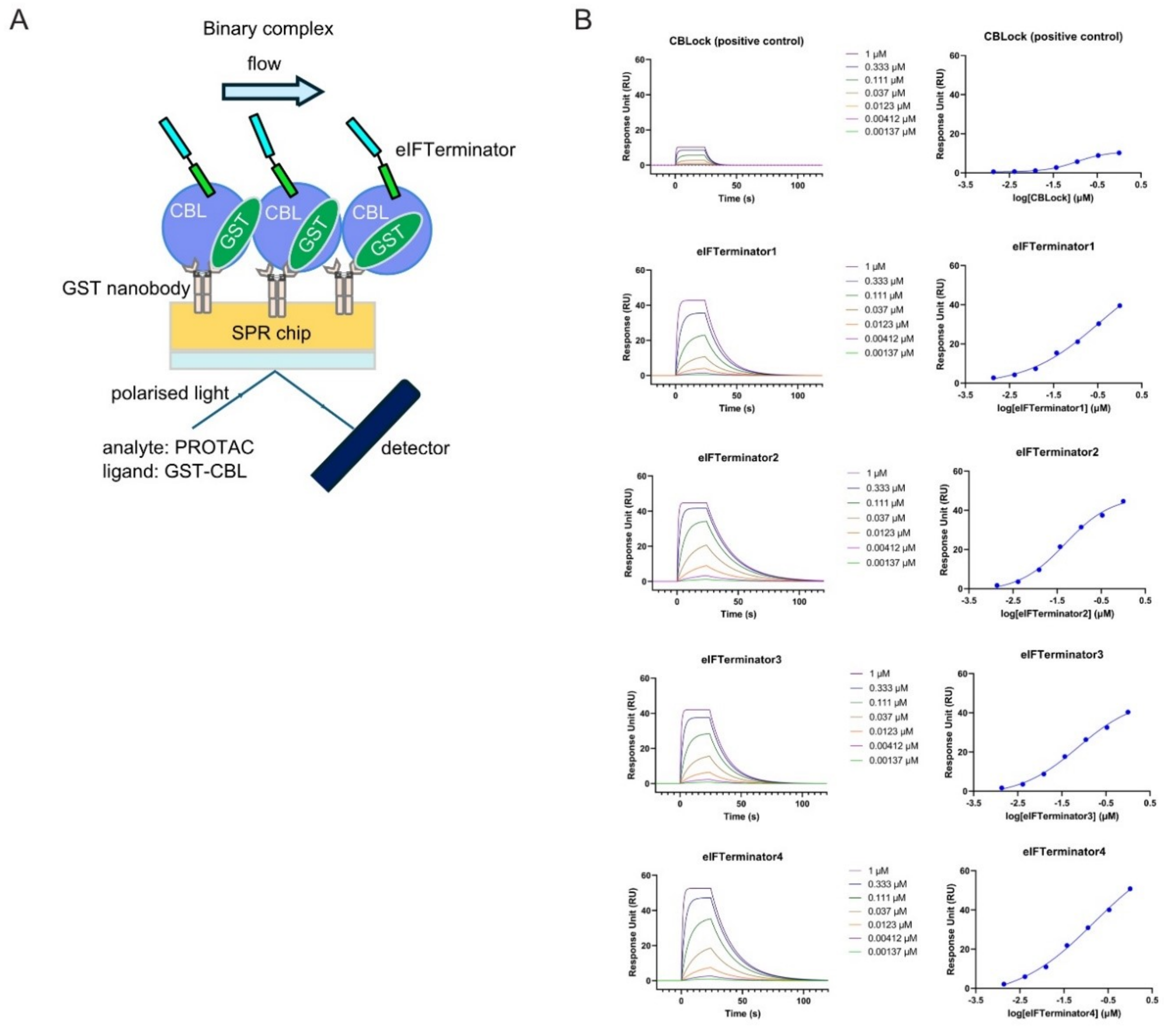

Supplementary Figure 3. Related to **Table 1**

A. Schematic illustration of the binary complex analysis by SPR. GST-tagged CBL was captured on to the SPR sensor chip with an anti-GST nanobody as the ligand, and individual eIFTerminators were flowed over the bound CBL.

B. Sensorgrams (left) and binding curves (right) showing a three-fold dilution series of the CBLock or eIFTerminator1-4 analytes (0.001372-1 μM) injected over immobilised CBL. Data are representative of three independent experiments.

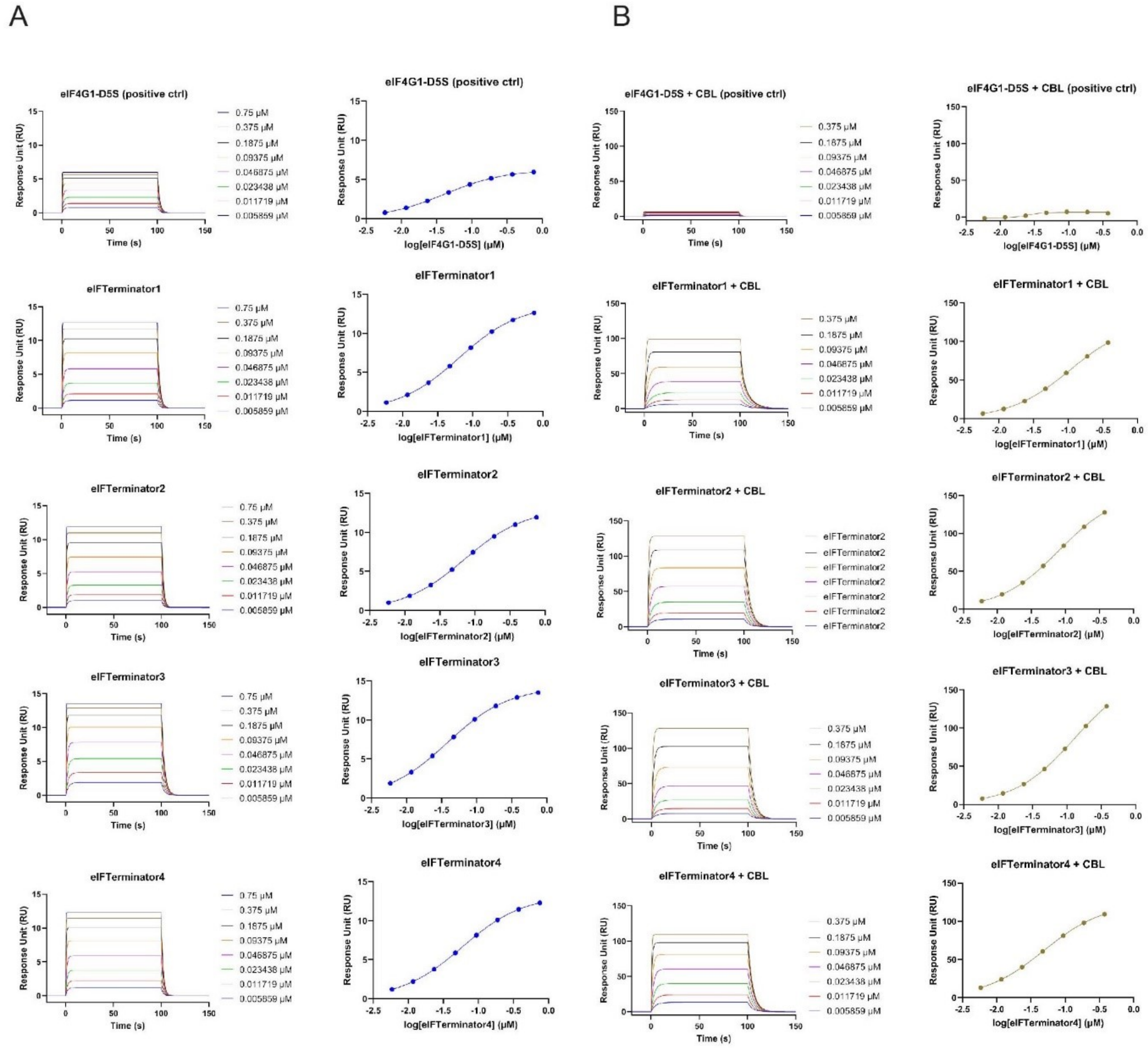

Supplementary Figure 4. Related to **Table 2**

A. Sensorgrams (left) and binding curves (right) showing a three-fold dilution series of the eIF4G1-D5S or eIFTerminator1-4 analytes (0.005859-0.75 μM) injected over immobilised eIF4E. Data are representative of three independent experiments.

B. Sensorgrams (left) and binding curves (right) showing a three-fold dilution series of the eIF4G1-D5S or eIFTerminator1-4 analytes (0.005859-0.375 μM) injected over immobilised eIF4E under a fixed concentration of 1 μM CBL. Data are representative of three independent experiments.

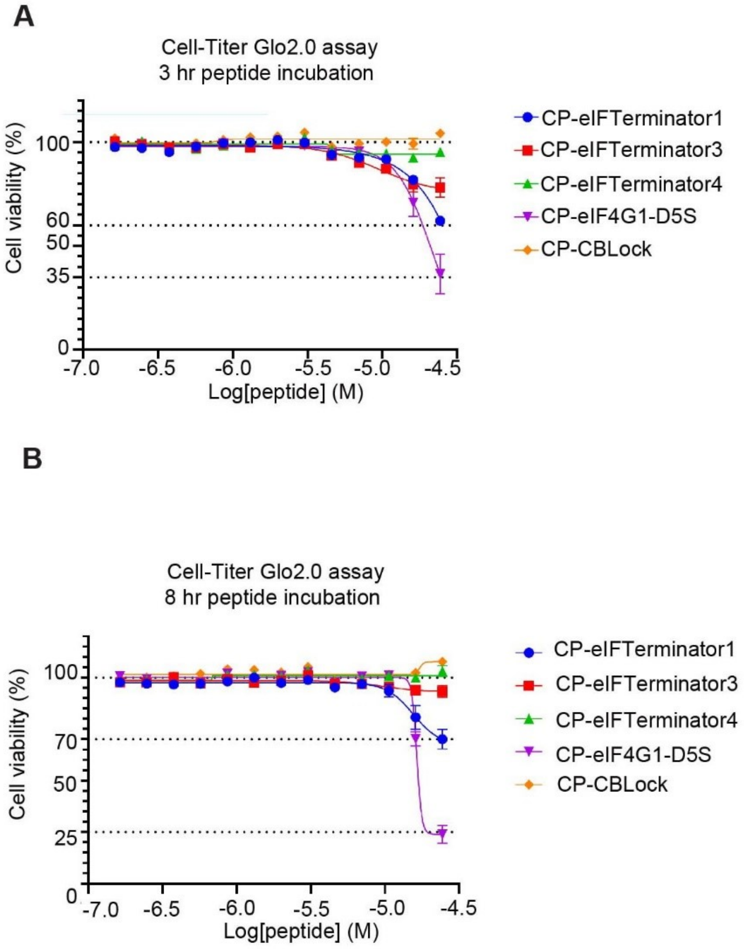

Supplementary Figure 5. Characterisation of cytotoxicity effects of CP-eIFTerminators and the individual ligands

A. Measurement of cell viability (%) in HeLa cells following 3-hr treatments with cell-penetrating peptides. A Cell Titer-Glo2.0 cell viability assay was performed to assess viability of HeLa cells following a 3-hr treatment with a dilution series of each CP-eIFTerminator or parental warhead (CP-CBLock or CP-eIF4G1-D5S). The concentration range of each peptide in the 13-point serial dilution was 0.16-24.4 µM. Data on CP-eIFTerminator2 are not shown as it was insoluble (n = 3, mean ± Standard error of the mean (SEM)).

B. Measurement of cell viability (%) in HeLa cells following 8 hr treatments with cell-penetrating peptides. A Cell Titer-Glo2.0 cell viability assay was performed to assess viability of HeLa cells following an 8-hr treatment with a dilution series of each CP-eIFTerminator, CP-CBLock, or CP-eIF4G1-D5S. The concentration range of each peptide in the 13-point serial dilution was 0.16-24.4 µM. CP-eIFTerminator2 was not

included in the analysis as it was insoluble (n = 3, mean ± Standard error of the mean (SEM)).

Methods

Plasmids

The following bacterial plasmids were generated in this study for protein purification. RSF-Duet His-TEV-eIF4E-GGS-CBLock (FP1), RSF-Duet His-TEV-eIF4E-$(GGS)_3$-CBLock (FP2), RSF-Duet His-TEV-CBLock-GGS-eIF4E (FP3), RSF-Duet His-TEV-CBLock-$(GGS)_3$-eIF4E (FP4), and RSF-Duet His-eIF4E. In addition, the following mammalian expression plasmids were generated: pLEX307 HA-eIF4E-$(GGS)_3$-CBLock (FP2) and pLEX307 HA-eIF4E.

pRSF1b UBE2D2, pGEX4T1 GST-TEV pTyr371-CBL (47–435), pGEX4T1 GST-TEV-GGSC-Ub, pGEX4T1 GST-TEV CBL TKBD (47–355), 8His-SUMO-Ulp1-CBL(47-435), pcDNA3.1 Myc-CBL, and pRSF-DUET1 GGS-6xHis UBA1 plasmids were generated and used in previous studies (2-7).

Antibodies and peptides

Table 3 lists the antibodies used in this study. The antibodies were diluted in TBS buffer containing 2.5% BSA.

Table 3 Antibodies

Name	Species	Supplier	Cat. No.	Stock concentration (µg/mL)	Dilution
Anti-CBL antibody [17/c-Cbl]	Mouse	Abcam	Ab309549	984	1 in 1000
Anti-CBLB antibody [246C5a]	Mouse	Abcam	Ab54362	100	1 in 1000
Anti-CBL-pY371 antibody	Rabbit	Eurogentec	custom made	1250	1 in 1000
Anti-His antibody	Mouse	Takara	631212	500	1 in 1000
Anti-puromycin antibody, clone 12D10	mouse	Merck	MABE343	500	1 in 1000

Beta-Actin Antibody (C4)	Mouse	Santa Cruz Biotechnology	sc-47778	200	1 in 1000
eIF4A1 Antibody	Rabbit	Cell Signaling Technology	2490S	88	1 in 1000
eIF4E (C46H6) Rabbit mAb	Rabbit	Cell Signaling Technology	2067S	54	1 in 1000
eIF4G (C65H5) Rabbit mAb	Rabbit	Cell Signaling Technology	2617S	254	1 in 1000
FITC Monoclonal Antibody	Mouse	ThermoFisher Scientific	MIF2901	5000	1 in 2000
HA-Tag (C29F4) Rabbit mAb	Rabbit	Cell Signaling Technology	3724S	66	1 in 1000
IRDye680LT goat anti-rabbit IgG secondary antibody	Goat	LI-COR Bioscience	926-68021	1000	1 in 5000
IRDye800CW goat anti-mouse IgG secondary antibody	Goat	LI-COR Bioscience	926-32210	1000	1 in 5000
Myc-Tag (9B11) Mouse mAb	Mouse	Cell Signaling Technology	2276	1000	1 in 1000
Ubiquitin antibody (P4D1)	mouse	Santa Cruz Biotechnology	sc-8017	200	1 in 1000

The peptides used for *in vitro* ubiquitination and SPR assays were purchased from Genscript; CBLock (N'-MTVEEMDSWIKSWDQR-C'), eIF4G1-D5S(N'-KKRYSREFLLGF-C'), eIFTerminator1 (N'-KKRYSREFLLGFGGSGMTVEEMDSWIKSWDQR-C'), eIFTerminator2 (N'-MTVEEMDSWIKSWDQRGGSGKKRYSREFLLGF-C'), eIFTerminator3 (N'-KKRYSREFLLGFGGSGGSGGSGMTVEEMDSWIKSWDQR-C'), eIFTerminator4 (N'-MTVEEMDSWIKSWDQRGGSGGSGGSGKKRYSREFLLGF-C'). All peptides were C-terminally amidated.

The cell-penetrating peptides used for cell-based assays were CP-CBLock (N'-RQIKIWFQNRRMKWKKGGSMTVEEMDSWIKSWDQR-C'), CP-eIF4G1-D5S (N'-RQIKIWFQNRRMKWKKGGSKKRYSREFLLGF-C'), CP-eIFTerminator1 (N'-RQIKIWFQNRRMKWKKGGSKKRYSREFLLGFGGSGMTVEEMDSWIKSWDQR-C'), CP-eIFTerminator2 (N'-RQIKIWFQNRRMKWKKGGSMTVEEMDSWIKSWDQRGGSGKKRYSREFLLGF-C'), CP-eIFTerminator3 (N'-RQIKIWFQNRRMKWKKGGSKKRYSREFLLGFGGSGGSGGSGMTVEEMDSWIKSWDQR-C'), CP-eIFTerminator4 (N'-

RQIKIWFQNRRMKWKKGGSMTVEEMDSWIKSWDQRGGSGGSGGSGKKRYSREFLLGF-C'). All peptides were amidated on the C-terminus, and CP-eIFTerminator1-4 were labelled with FITC on the C-terminus. CP-CBLock was purchased from Bioserve. CO-eIF4G1-D5S was bought from Generon. CP-eIFTerminator1 was obtained from Neobiotech. eIFTerminator2-4 were synthesised by Genscript.

Protein expression using bacterial plasmids

E. coli BL21(DE3) Gold cells (Agilent) were transformed with plasmids for protein expression. Colonies were grown on an agar plate with 50 μg/mL kanamycin, and a single colony was inoculated overnight in 4 mL LB supplemented with 50 μg/mL kanamycin at 37°C and 180 rpm. The starting culture was added to a baffled flask containing 1 L of warm LB with 50 μg/mL kanamycin on the following day. The flasks were placed in a shaker at 37°C and 200 rpm until the optical density (OD600nm) reached 0.6 – 1.0. The temperature was lowered to 20°C and 1 mL of 0.2 M Isopropyl β-D-1-thiogalactopyranoside (IPTG) was added to each flask. The cells were harvested on the following day by centrifugation. A Beckman JS-4.2 rotor was used to centrifuge cultures placed in 1L centrifuge bottles at 4°C, 4500 g for 15 min. The supernatant was removed, and the pellets were resuspended in wash buffer (25 mM Tris-HCl pH 7.6, 0.2 M NaCl, 30 mM imidazole, 5 mM β-mercaptoethanol). The resuspended cells were flash frozen after adding phenylmethanesulphonyfluoride (PMSF; final concentration: 2.5 mM) and lysozyme (final concentration: 1 mg/mL) and stored at -40°C.

Purification of CBLock-eIF4E fusion proteins (FP1-4)

Recombinant His-tagged proteins were expressed in *E. coli* BL21(DE3) Gold cells (Agilent) and purified using a two-step protocol. Econo-Pac gravity columns (Bio-Rad, Cat. No. 7321010) and high-density nickel resin (Agarose bead technology, Cat. No. 6BCL-QHNi-100) were used for affinity chromatography. The wash buffer for Ni^{2+}-purification comprised 25 mM Tris-HCl pH 7.6, 0.2 M NaCl, 30 mM imidazole, 5 mM β-mercaptoethanol. The elution buffer composition was the same except that the concentration of imidazole was increased to 300 mM. Following elution, the samples underwent dialysis overnight at 4 °C in 25 mM Tris-HCl pH 7.6, 0.2 M NaCl, and 5 mM β-mercaptoethanol.

Subsequently, gel filtration was performed on a Superdex 75 Increase 10/300 GL column (Cytiva) equilibrated in 25 mM Tris-HCl pH 7.6,150 mM NaCl, and 1 mM DTT using an AKTA Purifier (Cytiva).

SDS-PAGE

NuPAGE™ 4 -12% Bis-Tris 10, 12, or 15-well gels (Invitrogen™) were used to separate proteins by SDS-PAGE in 1x NuPAGE™ MES SDS running buffer. All proteins were mixed with NuPAGE™ loading dye (1x final concentration) and 100 mM DTT prior to loading. Quick Coomassie Stain (Neo Biotech) was used to visualise bands. The gels were imaged using an Epson Perfection V750 Pro (Epson) scanner or a LICOR Odyssey Infrared Imaging Scanner CLX (LICOR). Alternatively, membranes for western blot analysis were subject to immunoblotting.

Immunoblotting

SDS-PAGE was performed, and proteins were separated based on molecular weight. Following this, the proteins were transferred from the SDS-PAGE gel onto a nitrocellulose membrane from a Trans-Blot Turbo RTA Mini 0.2 µm nitrocellulose transfer kit (Bio-Rad) using the Trans-Blot Turbo Transfer system (Bio-Rad) following the manufacturer’s protocol. Subsequently, the membrane was washed with 1x TBS. After blocking the membrane with 5% BSA in TBS for 1 hr at RT, it was incubated with primary antibody at a dilution as described in **Table 3** overnight at 4°C. Actin was used as loading control. The membrane was washed three times with TBS-T, and appropriate secondary antibodies **Table 3** were incubated for 1 hr. The secondary antibodies were removed after three washes with TBS-T. The membrane was dried and scanned using a LICOR Odyssey CLX imager system after a final wash with TBS.

***In vitro* ubiquitination assay**

Peptides and purified proteins were used to perform *in vitro* ubiquitination assays. The final assay buffer comprised 50 mM Tris-HCl (pH 7.6), 50 mM NaCl, 5 mM $MgCl_2$ and 5 mM ATP. All reactions were performed at RT.

In vitro ubiquitination assay of eIF4E-CBLock fusion proteins (FP1-4)

The final concentrations of proteins in the assay were: 50 μM Ub, 5 μM UBE2D2, 1 μM UBA1, 1 μM substrate (FP1-4 or His-eIF4E), and 0.1 μM pTyr371-CBL. A 10x master mix without Ub was prepared first in an eppendorf tube. A fraction of this sample was transferred to an eppendorf with 4x NuPAGE™ LDS sample buffer loading dye mixed with 250 mM DTT and was treated as the 0 min time point. Fluorescently labelled Ub was added to the master mix to initiate the reaction. Fractions of the master mix were transferred to eppendorf tubes with loading dye to collect the 6 min time point samples. The samples were then analysed by SDS-PAGE. The gels were imaged using a LICOR Odyssey Infrared Imaging Scanner CLX for fluorescence detection. Subsequently, the gels were stained with Quick Coomassie Stain and imaged using an Epson Perfection V750 Pro scanner.

The *in vitro* ubiquitination assay was repeated for western blotting. 0 min and 6 min time points were taken, and the samples were blotted with an anti-eIF4E antibody and IRDye800CW goat anti-mouse IgG secondary antibody to visualise eIF4E bands.

In vitro ubiquitination assay with eIFTerminator1-4

eIF4E ubiquitination was examined in the presence of eIFTerminator1-4 in *in vitro* ubiquitination assays. Briefly, 1 μM His-eIF4E, 50 μM fluorescent Ub, 5 μM UBE2D2, 1 μM UBA1, 200 nM pTyr371-CBL, and 0.75 μM of eIFTerminator1-4 were included in the 1x reaction mix.

The samples were run on SDS-PAGE. The gels were scanned with a LICOR Odyssey Infrared Imaging Scanner CLX for fluorescence detection, and subsequently Coomassie-stained for imaging with an Epson Perfection V750 Pro scanner.

Mass spectrometry for ubiquitination site analysis

The bands of interest from Coomassie-stained gels were processed for mass spectrometry analysis. The excised gel fragments were washed twice with 50 mM ammonium bicarbonate as well as 50 mM ammonium bicarbonate with 50% acetonitrile. Proteins in the gels were reduced with 10 mM DTT at 54 °C for 30 min and alkylated with 55 mM

iodoacetamide at RT for 45 min. Gels were washed again and dehydrated with acetonitrile prior to drying with SpeedVac. Trypsin Gold (Promega) diluted in 25 mM ammonium bicarbonate was added to the gel at 5 μg/mL and incubated for 12 hr at 35 °C. Digested peptides were extracted with 50% acetronitrile/1% trifluoroacetic acid and dried in a SpeedVac. Peptides were resuspended in 1% trifluoroacetic acid and desalted using StageTip (Cell Signaling Technology) as described by Rappsilber, Mann (8). Trypsinised peptides were separated by nanoscale C18 reverse-phase chromatography using an EASY-nLC II 1200 (Thermo Scientific) coupled to an Orbitrap Q-Exactive HF mass spectrometer (Thermo Scientific).

The mass spectrometer was operated in positive mode. A resolution of 60,000 at 250 Th was used for a full scan, and the top ten intense ions in the full mass spectra were isolated for fragmentation for a maximum of 75 ms with a target of 100,000 ions. The fragmentation spectra were then read at the resolution of 15,000 at 250 Th. Ions already selected for MS2 were dynamically excluded for 20 s. Mass spectrometry data were acquired using the XCalibur™ software (Thermo Fisher Scientific).

Raw data were processed with MaxQuant software (9) version 1.6.14.0 and searched with the Andromeda search engine (10), querying SwissProt (11), *Escherichia coli* (4934 entries), and a fasta file containing the sequence of Ub and FP1-4.

First and main searches were performed with precursor mass tolerances of 20 ppm and 4.5 ppm, respectively, and an MS/MS tolerance of 20 ppm. The minimum peptide length was set to six amino acids and specificity for trypsin cleavage was required. Cysteine carbamidomethylation was set as a fixed modification, whereas Methionine oxidation, di-Gly-Lysine and N-terminal acetylation were specified as variable modifications. The peptide, protein, and site false discovery rate (FDR) were set to 1%. For ubiquitinated peptide analysis, the MaxQuant output GlyGly (K)Sites.txt file was imported into Perseus software version 1.6.2.3 (12). Reverse and potential contaminant flagged peptides were removed from the GlyGly (K)Sites.txt file, and only ubiquitinated peptides having: “Score diff” greater than 5, and a “Localisation probability” higher than 0.75 were included in the heatmap.

Mass spectrometry analysis for quantitative proteome analysis

HeLa cells were lysed in buffer consisting of 50 mM Tris-HCl (pH 7.5), 150 mM NaCl, 1% Triton-X, 1% SDS, 1 μL benzonase and an EDTA-free Protease Inhibitor Tablet. Extracted

proteins were reduced using 10 mM DTT for 30 min at 54°C and subsequently alkylated using 55 mM iodoacetamide at 25°C for 1 hr in the dark. Alkylated proteins were mixed with conditioned magnetic hydroxyl beads (Resyn Bioscience) and were left to aggregate for 30 min in 70% acetonitrile. Samples were then transferred to a magnetic rack, and the beads were washed in 70% and 95% acetonitrile, discarding the supernatant during the wash. Protein beads aggregates were resuspended in 100 mM ammonium bicarbonate, and trypsin was added with an enzyme: substrate ratio of 1:200 after an overnight incubation on a shaker at 35°C and 1000 rpm. Samples were finally desalted using StageTip (8).

The digested peptides were separated by nanoscale C18 reverse-phase liquid chromatography using an EASY-nLC II 1200 coupled to an Orbitrap Fusion Lumos mass spectrometer (Thermo Scientific) for the proteome analysis. A binary gradient was used for elution at a flow rate of 300 nL/min, into a 50 cm fused silica emitter (New Objective) packed in-house with ReproSil-Pur C18-AQ, 1.9 μm resin (Dr Maisch GmbH). Packed emitter was kept at 50 °C by means of a column oven (Sonation) integrated into the nanoelectrospray ion source (Thermo Scientific). Active Background Ion Reduction Device (ABIRD) was used to reduce air contaminants signal level.

Proteome analysis was carried out over a period of 125 min in positive ion mode using data-independent acquisition (DIA). A full scan (FT-MS) over mass range of 340–1050 m/z was acquired at 60,000 resolution at 200 m/z. The target value was 3,000,000 ions for a maximum injection time of 54 ms. Higher energy collisional dissociation fragmentation spectra were recorded at 30000 resolution and 200 m/z. Every precursor was fragmented using 28 consecutive windows with 25 Da width. This allowed for a 0.5 m/z overlap, covering a mass range from 350 to 1023 m/z. All ions were fragmented with normalised collision energy of 28 for a maximum injection time of 54 ms, or a target value of 3e6 ions.

The Proteome Raw data were processed with Spectronaut version 20.1 (13) using directDIA analysis querying Uniprot (14). This allowed up to two missed cleavage sites and minimum peptide length to 7. Carbamidomethylation of cysteine was set as fixed modification and methionine oxidation and N-terminal acetylation were specified as variable modifications. Minor peptide grouping was set to “Modified Sequence”, and Major and Minor Group Quantity were set to “Sum peptide quantity” and “Median precursor quantity”, respectively. Protein and precursor PEP cutoff was set to 0.05, single hit proteins were excluded from dataset and all

other parameter in were left to default values. Spectronaut results for protein and peptide were exported and analysed using Perseus software version 1.6.14.0 (12).

A Fisher exact test was applied using Perseus to identify significantly enriched KEGG pathways associated with increased protein fold change in the no PROTAC controls (12). Categories were deemed significantly enriched if they passed the threshold value of 0.01 using Benjamini-Hochberg FDR for truncation.

Surface Plasmon Resonance (SPR)

A Biacore T200 was used to study binding affinity and binding kinetics. Data were analysed using Cytiva Biacore™ Insight Evaluation Software 5.0.18.22102. Experiments were performed at 25°C, and sensorgrams were processed by reference subtraction from blank. Peptides were studied as analytes, excluding the warhead that does not bind to the immobilised ligand.

Studying the interaction between PROTAC and GST-CBL

Anti-GST nanobodies (Chromotec) were amine-coupled to a CM5 chip (Cytiva). Briefly, after priming the surfaces of all four channels with 1x HBS-EP+ buffer (Cytiva), the surface was activated with NHS and EDC (Cytiva) by injecting them individually for 5 µL/min for 420 s. The anti-GST nanobody was injected at a flow rate of 5 µL /min for 420 s. 1 M ethanolamine was added for 420 s at a flow rate of 10 µL/min, resulting in a final Response Unit (RU) of approximately 750.

The GST nanobody was used to capture GST-tagged proteins or GST tag. His-GST-pTy371-CBL was diluted to 0.1 mg/mL in running buffer (200 mM NaCl, 25 mM Tris-HCl pH 7.6, 1 mM DTT, 100 µg/mL BSA, and 0.005% Tween20) and was captured at a surface density of 500 - 840 RU in Fc2 and Fc4 as duplicate samples. Purified 5 µg/mL GST was captured as a control and captured on Fc1 and Fc3 as duplicates. Titrations of eIFTerminator1-4 and CBLock were prepared in running buffer at concentrations ranging from 5.08 x 10^{-5} -3 µM in a 3-fold serial dilution. Two identical titrations were made per peptide that was studied. The buffer was filtered with 0.22 µm filter paper and a NALGENE reusable bottle top filter prior to use.

The peptides were reconstituted in running buffer and filtered using Costar Spin-X centrifuge tube filters 0.22 μM by centrifugation at 15,871 g for 10 min at 4°C to remove precipitation. During the run, 10 washing cycles were done prior to analyte injection, and buffer control runs were performed every 6th cycle of measurements. The experiment was performed at a flow rate of 30 μL/min, with a 24 s contact time and a 180 s dissociation time. Data were analysed by kinetic-state affinity analysis using Cytiva Biacore™ Insight Evaluation Software. Sensorgrams for figures were made using GraphPad Prism 9.5.1.

Studying the interactions between eIF4E and eIFTerminator1-4

His-eIF4E protein was immobilised on Cytiva's sensor chip CM5 using amine-coupling. The chip was docked and primed in running buffer (50 mM HEPES pH 7.5, 100 mM NaCl, 1 mM TCEP, 0.05% Tween20). Cytiva's amine-coupling kit was used to capture the ligand. Briefly, 0.4 M EDC and 0.1 M NHS from the kit were mixed 50:50 and injected to activate the chip surface for 300 s at 10 μL/min. His-eIF4E was prepared for amine coupling as described previously (15). After achieving a capture level of approximately 120 RU, the remaining binding sites were blocked with ethanolamine hydrochloride-NaOH (pH 8.5) from the kit for 420 s at a flow rate of 10 μL/min. Reference surfaces were prepared in duplicates per chip.

Multi-cycle experiments were performed to gain steady-state binding affinity of eIFTerminator1-4 to His-eIF4E. At a flow rate of 30 μL/min, 20 startups with a 30 s contact time and a 30 s dissociation time were done prior to sample injection. An 8-point, 1 in 2 dilution series of eIFTerminator1-4 or eIF4G1-D5S peptide was injected (0.005859 – 0.75 μM) with a 100 s contact time and a 250 s dissociation time at a flow rate of 30 μL/min. The peptides were dissolved in running buffer and filtered using Coster Spin-X 0.22 μM centrifuge tube filters as described above. Three buffer only samples were injected per titration as blanks.

Studying ternary complex cooperativity

The cooperativity of eIFTerminators was studied concomitantly with the binding study between PROTAC and eIF4E in binary condition. A 1 μM fixed concentration of CBL (47-435) or CBL-B (36-427) was mixed with 8-point, 1 in 2 dilution series of

eIFTerminator1-4 that varied between 0.005859 – 0.375 μM. Three buffer only samples were included per titration as blanks. Triplicates of CBL (47-435) or CBL-B (36-427) only injections were also included to test substrate binding to the chip.

Mammalian cell culture conditions

HeLa cells were purchased from the German Collection of Microorganisms and Cell Cultures GmbH and cultured in 10% Fetal Bovine Serum and 5 mL Penicillin Streptomycin in RPMI medium 1640 for all experiments except the Cell-Titer Glo2.0 and AlphaLISA assays. HeLa cells used in these assays were obtained from AstraZeneca's Cell Banking System, originally sourced from the European Collection of Authenticated Cell Cultures (ECACC). The cell culture medium consisted of DMEM – high glucose supplemented with 10% FBS and 5 mL GlutaMAX (ThermoFisher Scientific). TrypLE^{TM} Express Enzyme (Gibco^{TM}) and PBS were used to split cells. HEK293 cells were purchased from the ECACC and cultured in 5 mL L-glutamine, 600 μL Gentamycin, 5 mL Penicillin Streptomycin, and 10% FBS in Dulbecco's Modified Eagle's Medium (DMEM). Lenti-X HEK293T (Takara) cells used for virus expansion were cultured in the same medium. U-2 OS cells were purchased from the American Type Culture Collection (ATCC) and were also maintained in the same culture conditions.

Cells were discarded when the passage number reached 20. 1x of pre-warmed 2.5% Trypsin (10x) was used to passage cells unless otherwise stated. Cell cultures were subject to regular mycoplasma testing and authenticated by STR fingerprint analysis.

Generation of stable cell lines

The DNA sequences encoding for HA-eIF4E-(GGS)3-CBLock or HA-eIF4E were inserted into NheI and EcoRV-digested pLEX307 plasmids (Addgene 41392) to generate pLEX307 HA-eIF4E-(GGS)3-CBLock or pLEX307 HA-eIF4E.

1.5 x 10^6 Lenti-X HEK293T cells were plated on the first day in 10 cm dishes. Lenti-X HEK293T cells were transfected with three lentiviral plasmids the next day. The plasmids were mixed at a 4:3:1 mass ratio of pLEX307:psPAX2:pMD2.G (10 μg pLEX307:7.5 μg

psPAX2: 2.5 µg pMD2.G). 20 µg DNA was mixed with 1.2 mL Opti-MEM, while 1.2 mL Opti-MEM was mixed with 50 µL Lipofectamine2000 and incubated for 5 min. The two tubes were mixed, and the DNA-liposome complex was added to fresh 10 mL culture medium 20 min later. The medium was replaced with 12 mL fresh medium 12-16 hr after transfection. The WT HEK293 cells to be transduced were seeded in a 6-well plate on the same day at 4 x 10^6 cells/mL, reaching confluency in three days. Viruses were collected 24-36 hr post media change. The media were centrifuged for 5 min at 260 g and subsequently filtered with 0.45 µm filters to remove Lenti-X HEK293T cells. Polybrene was added to the medium-containing virus (final concentration: 8 µg/mL). The medium from the target HEK293 cells was aspirated and replaced with the polybrene and virus-containing medium to allow lentiviral transduction.

Medium of the transduced cells was replaced 24 hr later, and cells were grown in medium supplemented with puromycin for the next three days for selection. The cells were subsequently aliquoted and frozen in culture medium containing 10% DMSO.

Western blot procedures for cell-based assays

Three washes with 1 mL PBS were performed before harvesting cells with a cell lifter. The cells were frozen in liquid nitrogen and stored at -70°C. The lysis buffer comprised 50 mM Tris-HCl (pH 7.5), 150 mM NaCl, 1% Triton-X, 1% SDS, 1 µL benzonase and one tablet of the Pierce™ Protease Inhibitor Tablets, EDTA-free (ThermoFisher Sceintific) in PBS except for HA-tag and Myc-tag IP. As for HA-tag and Myc-tag IP, the lysis buffer consisted of 50 mM Tris-HCl (pH 7.5), 150 mM NaCl, 1 mM EDTA, 1% IGEPAL CA-630, 10% glycerol, 20 µM PMSF, 1 mM DTT, and a single Protease Inhibitor Tablet in PBS.

The cells were lysed in 35 µL lysis buffer at 4°C by vortexing every 10 min over a period of 90 min. Protein concentration in the cell lysate was measured by Bradford assay. Lysates were immunoprecipitated in addition to inputs when appropriate. SDS-PAGE and immunoblotting analysis were performed subsequently.

Time course of Tyr371 phosphorylation of CBL and Myc-IP

HEK293 cells were seeded in 10 cm dishes at 2.2 x 10^6 cells per dish and kept overnight at 37°C, 5% CO_2. Myc-CBL was overexpressed on the subsequent day. Two tubes

were prepared for transfection. 15 µL Lipofectamine2000 and 500 µL Opti-MEM were incubated for 5 min in the first Eppendorf tube. In the second Eppendorf tube, 6 µg Myc-CBL construct and 500 µL Opti-MEM were incubated for 5 min. The two tubes were mixed and kept at RT for 25-30 min. The mix was subsequently added to cell culture medium. The medium was replenished on the next day, and cells were stimulated with 50 ng/mL EGF (Merck, Cat. No.: E9644) over the course of 2 hr. The cells were harvested for Myc-tag immunoprecipitation (Myc-IP) and immunoblot analysis. After solubilising the harvested pellets in lysis buffer, 0.5 mg protein per sample was incubated with 25 µL Myc beads (Proteintech) overnight and kept in the cold room. The samples were washed three times with lysis buffer and eluted with 30 µL 2x LDS. To achieve this, a 4x LDS mix that consisted of 900 µL LDS and 100 µL DTT mix was prepared. Following this, 2x LDS was made by mixing 100 µL of 4x LDS dye with 100 µL water. Western blotting was performed to analyse the inputs and Myc IP samples. Anti-Myc, anti-actin, and anti-CBL-pY371 antibodies were used as primary antibodies. Goat IRDye800CW goat anti-mouse IgG secondary antibody and IRDye680LT goat anti-rabbit secondary IgG were used as secondary antibodies.

HA-tag IP of HA-FP2 under denaturing condition

HA-FP2 or HA-eIF4E were stably expressed in HEK293 cells. Cells were stimulated with 100 ng/mL EGF for 3 hr prior to harvest.

To prepare the lysate to be immunoprecipitated, the pellets were thawed on ice and 75 µL RIPA lysis buffer was added. The RIPA buffer consisted of 50 mM Tris-HCl (pH7.5), 250 mM KCl, 10% glycerol, 0.25% IGPAL CA-630, 2 mM $MgCl_2$, protease inhibitor, 1 µL benzonase, and 1% sodium dodecyl sulphate. Samples were boiled for 10 min at 92°C and subsequently diluted 1 in 10 with RIPA buffer to achieve a total volume of 750 µL per sample containing 0.1% SDS. The tubes were centrifuged at 15,871 g for 10 min at 4°C, and the supernatant was transferred to a new tube for a Bradford assay. Each sample was topped up with lysis buffer so that each 1.5 mL Eppendorf tube contained 1 mg protein of the cell lysate in 1 mL buffer. HA Pierce anti-HA magnetic beads (Thermo Scientific) were added and the lysate was incubated with the beads for 30 min at RT to perform immunoprecipitation. After 30 min, the supernatant was removed, and the tubes were placed on a magnetic stand for three washes with 1 mL wash buffer. The wash buffer contained 50 mM Tris-HCl (pH7.5), 250 mM KCl, 10% glycerol, 0.25% IGPAL CA-630, and 2 mM

$MgCl_2$. After the liquid was removed, 30 μL 2x LDS mix (prepared as described above) was used to elute the IP samples. The samples were boiled at 92°C for 10 min and 28 μL were loaded on a 4-12% Bis-Tris SDS-PAGE gel, along with the whole cell lysates that were prepared in 75 μL RIPA buffer. The gel was subsequently transferred and western blotted. Antibodies included anti-actin, anti-HA, anti-K48 antibodies as primaries, as well as IRDye800CW goat anti-mouse IgG secondary antibody and IRDye680LT goat anti-rabbit secondary IgG.

CellTiter-Glo2.0 Cell Viability Assay

Cells were seeded onto a Greiner 384 small volume plate using a Multidrop™ Combi Reagent Dispenser (Thermo Scientific). The seeding density was 2000 cells/well in a volume of 15 μL per well. After incubating the 384-well plate overnight at 37°C, 5% CO_2, the medium on the plate was removed using a Bluewasher (Blue Cat Bio). 15 μL of fresh medium supplemented with 100 ng/mL EGF was added to each well. The cell-penetrating peptides were dispensed using a Tecan D300. A 13-point serial dilution series of 0.163 μM – 24.4 μM of each peptide dissolved in DMSO was dispensed with the highest concentration of DMSO at less than 0.5%. Three replicates were run per condition. The assay plate was incubated for 3 hr or 8 hr at 37°C, 5% CO_2. Following this, the liquid on the assay plate was removed using the Bluewasher. 10 μL of the 1:1 mix of PBS and the CellTiter-Glo2.0 reagent from CellTiter-Glo 2.0 Cell Viability Assay kit (Promega) was dispensed using the Multidrop to individual wells. The plate was read using a PHERAstar FSX (BMG Labtech). Raw data were exported from the Pherastar as a Microsoft Excel file and analysed in Excel, and the processed data were plotted in GraphPad Prism v9.5.1.

Comparison of CP-eIFTerminator peptides and warheads in HeLa cells

HeLa cells were seeded in 6 cm dishes at a cell density of 1.5 x10^6 per dish. After overnight incubation at 37°C, 5% CO_2, cells were treated with 100 ng/mL along with 10 μM of respective cell-penetrating peptides. Peptides were reconstituted in water immediately before addition to the cell culture. An equal volume of water was added as a negative control. Cells were harvested 3 hr after peptide incubation at 37°C, 5% CO_2 for western blot analysis.

Anti-actin, anti-eIF4EA, anti-eIF4E, anti-eIF4G, and anti-FITC antibodies, as well as IRDye800CW goat anti-mouse IgG secondary antibody and IRDye680LT goat anti-rabbit secondary IgG in **Table 3** were used for Western blot.

Time course of CP-eIFTerminator4 in HeLa cells

0.5 x 10^6 HeLa cells were seeded in 35 mm dishes. A time course experiment was initiated after overnight incubation at 37°C, 5% CO_2. For each time point (0.5, 3, 6, 12, 24 hr), cells were treated with fresh medium containing 100 ng/mL EGF along with 20 μM CP-eIFTerminator4 or water. All dishes were harvested at the same time, after varying incubations with the peptide.

Titration of CP-eIFTerminator4 in HeLa and U-2 OS cells

Cells were seeded in 35 mm culture dishes at the density of 1.5 x 10^6 cells/mL and kept overnight at 37°C, 5% CO_2. The medium was replaced with fresh medium supplemented with 100 ng/mL EGF. Immediately after this, 2.5 μM, 10 μM, or 50 μM CP-eIFTerminator4 was added to U-2 OS cells. 5 μM, 10 μM, or 20 μM CP-eIFTerminator4 was added in the case of HeLa cells. The cells were incubated at 37°C, 5% CO_2 for 3 hr prior to harvest. Anti-actin, anti-eIF4E, and anti-FITC antibodies were used as primary antibodies. IRDye800CW goat anti-mouse IgG secondary antibody was used to target the primary antibodies.

Degradation pathway inhibition in HeLa cells

The experiment where cells were treated with a proteasomal or lysosomal inhibitor was performed by first seeding HeLa cells in 35 mm culture dishes at a density of 1.5 x 10^6 cells/mL. Cells were treated with 100 nM carfilzomib (Cell Signaling Technology, Cat. No.: 15022) or 50 μM chloroquine (Cell Signaling Technology, Cat. No.: 14774S) and incubated for 4 hr at 37 °C, 5% CO_2. Fresh medium was supplemented with 100 ng/mL EGF 4 hr later, together with fresh 100 nM carfilzomib or 50 μM chloroquine and 20 μM CP-eIFTerminator4. Equal volumes of water were added instead of CP-eIFTerminator4 in the negative control samples. Cells were harvested 3 hr post CP-eIFTerminator4 treatment. The cell lysates were analysed by western blot. Primary antibodies used were anti-actin, anti-eIF4E, and anti-FITC antibodies. Secondary antibodies were IRDye800CW goat anti-mouse IgG secondary antibodies.

Short interfering RNA transfection

HeLa cells seeded in 6 cm dishes with 50% confluency were transfected with siRNA against *CBL* (Horizon, Cat. No.: L-003003-00-0005), *CBL-B* (Horizon, Cat. No.: L-003004-00-0005), and *EIF4E* (Horizon, Cat. No.: L-003884-00-0005), 16 hr after seeding at 0.4 x 10^6 cells per dish. The final concentration of each siRNA was 25 nM. A pair of Eppendorf tubes was prepared, one with 10 μL DharmaFECT 1 Transfection Reagent (Horizon, Cat. No.: T-2001-02) mixed with 190 μL Opti-MEM (Fisher Scientific, Cat. No.: 31985047) and another with 2.5 μL 20 μM siRNA with 7.5 uL nuclease free water (Horizon, Cat. No.: B-003000-WB-100) and 190 uL warm Opti-MEM transfection reagent. In the case of double knock-down, 2.5 μL *CBL* siRNA was mixed with 2.5 μL *CBL-B* siRNA and 5 μL nuclease free water in 190 uL Opti-MEM. The tubes were combined 5 min later, gently mixed and left for 20 min. The 400 μL mix was subsequently added to the 6 cm dish with 1.6 mL complete antibiotic and FBS-free medium. The medium was removed and replenished with fresh medium containing FBS 2 hr post transfection. Cells were grown for 75 hr in total for *CBL* and *CBL-B* siRNA experiments, and 20 μM CP-eIFTerminator4 was added 3 hr prior to harvest. HeLa cells were grown for 96 hr after transfection in the case of eIF4E.

AlphaLISA

HeLa cells were seeded onto a Greiner 384-well plate using a Multidrop. The seeding volume was 15 μL and the density was 2000 cells/well. The cell plate was incubated at 37°C,

5% CO_2 overnight. The medium was removed with a Bluewasher, and a Multidrop was used to replenish fresh medium supplemented with 100 ng/mL EGF (15 µL per well) approximately 16 hr post seeding. A 10-point titration of CP-eIFTerminator4 in concentrations ranging from 0.16-30 µM were dispensed using the Echo 650 Series Acoustic Liquid Handler (Beckman Coulter, Cat. No.: 001-16079), with three replicates per condition. A 1 mM stock concentration of CP-eIFTerminator4 was placed on the Echo 384LDV Qualified Source Plate (Beckman Coulter, Cat. No.: 001-12782) and the 384-well assay plate was selected as the destination plate for protocol execution. As a negative control, 30 µM CP-eIFTerminator4 was added to three wells where cells were not seeded but incubated with 15 µL medium. The assay plate was incubated at 37°C, 5% CO_2 for 3 hr. After removing medium with the Bluewasher, 5 µL lysis buffer from the AlphaLISA kit (Revvity, Cat. No.: ALSU-TEIF4-A500) was added to each well. The acceptor mix was prepared by combining the kit's RB1, RB2, and pre-warmed activation buffer following the manufacturer's instructions. This was further diluted in PBS (1 in 2 dilution).

The plate was centrifuged at 1000 rpm for 1 min and incubated at RT for an hour while avoiding exposure to light. Subsequently, 2.5 µL of the diluted donor beads were added to the plate using an electronic liquid handler. The donor bead mix was prepared by first diluting the donor beads 50-fold in dilution buffer, and further diluting in PBS in a ratio of 1 in 2. Following this step, the plate was centrifuged at 1000 rpm for 1 min. A black seal was placed on the plate and the assay plate was incubated overnight at RT. An Envision plate reader (Envision) was used to read the plate. Data exported as an Excel file were plotted using GraphPad Prism v9.5.1.

Puromycin incorporation assay for monitoring protein synthesis

HeLa cells were seeded in 3.5 mm dishes at a seeding density of 0.5 x 10^6 per dish. 100 ng/mL EGF in fresh cell culture medium was added to all culture dishes on the next day, and either water or 20 µM CP-eIFTerminator4 was added for 0 hr and 8 hr time points. Cells were treated with a final concentration of 1.158 µM puromycin 1 hr before harvest for the 8 hr samples. In the case of 0 hr time point samples, puromycin was added concomitantly with EGF and CP-eIFTerminator4 or water, and cells were harvested immediately after. The cell lysates were analysed with anti-actin, anti-eIF4E, anti-FITC and anti-puromycin antibodies

and IRDye800CW goat anti-mouse IgG secondary antibodies to visualise the nascent peptides that incorporated puromycin with or without CP-eIFTermiantor4 treatment.

Pymol

Modelling of FP1-4 and eIFTerminator1-4 design was performed using Pymol based on existing crystal structures.

Quantification and statistical analysis

GraphPad Prism v9.5.1 was used for statistical analyses on Cell Titer-Glo2.0 and AlphaLISA assays. Excel was used to perform all other statistical analyses. Image Studio Lite Ver 5.2 was used for quantification of immunoblot bands.

Supplementary references

1. Tomoo K, Shen X, Okabe K, Nozoe Y, Fukuhara S, Morino S, et al. Crystal structures of 7-methylguanosine 5'-triphosphate (m(7)GTP)- and P(1)-7-methylguanosine-P(3)-adenosine-5',5'-triphosphate (m(7)GpppA)-bound human full-length eukaryotic initiation factor 4E: biological importance of the C-terminal flexible region. Biochem J. 2002;362(Pt 3):539-44.
2. Nakasone MA, Majorek KA, Gabrielsen M, Sibbet GJ, Smith BO, Huang DT. Structure of UBE2K-Ub/E3/polyUb reveals mechanisms of K48-linked Ub chain extension. Nat Chem Biol. 2022;18(4):422-31.
3. Ahmed SF, Anand J, Zhang W, Buetow L, Rishi L, Mitchell L, et al. Locking CBL TKBD in its native conformation presents a novel therapeutic opportunity in mutant CBL-dependent leukemia. Mol Ther. 2025.
4. Ahmed SF, Buetow L, Gabrielsen M, Lilla S, Sibbet GJ, Sumpton D, et al. E3 ligase-inactivation rewires CBL interactome to elicit oncogenesis by hijacking RTK-CBL-CIN85 axis. Oncogene. 2021;40(12):2149-64.
5. Dou H, Buetow L, Hock A, Sibbet GJ, Vousden KH, Huang DT. Structural basis for autoinhibition and phosphorylation-dependent activation of c-Cbl. Nat Struct Mol Biol. 2012;19(2):184-92.
6. Mfuh AM, Boerth JA, Bommakanti G, Chan C, Chinn AJ, Code E, et al. Discovery, Optimization, and Biological Evaluation of Arylpyridones as Cbl-b Inhibitors. J Med Chem. 2024;67(2):1500-12.
7. Magnussen HM, Ahmed SF, Sibbet GJ, Hristova VA, Nomura K, Hock AK, et al. Structural basis for DNA damage-induced phosphoregulation of MDM2 RING domain. Nat Commun. 2020;11(1):2094.
8. Rappsilber J, Mann M, Ishihama Y. Protocol for micro-purification, enrichment, pre-fractionation and storage of peptides for proteomics using StageTips. Nat Protoc. 2007;2(8):1896-906.

9. Cox J, Mann M. MaxQuant enables high peptide identification rates, individualized p.p.b.-range mass accuracies and proteome-wide protein quantification. Nat Biotechnol. 2008;26(12):1367-72.
10. Cox J, Neuhauser N, Michalski A, Scheltema RA, Olsen JV, Mann M. Andromeda: a peptide search engine integrated into the MaxQuant environment. J Proteome Res. 2011;10(4):1794-805.
11. UniProt C. UniProt: a worldwide hub of protein knowledge 2019 [updated Jan 8PMC6323992]. 2018/11/06:[D506-D15]. Available from: https://www.ncbi.nlm.nih.gov/pubmed/30395287.
12. Tyanova S, Temu T, Sinitcyn P, Carlson A, Hein MY, Geiger T, et al. The Perseus computational platform for comprehensive analysis of (prote)omics data. Nat Methods. 2016;13(9):731-40.
13. Bruderer R, Bernhardt OM, Gandhi T, Miladinovic SM, Cheng LY, Messner S, et al. Extending the limits of quantitative proteome profiling with data-independent acquisition and application to acetaminophen-treated three-dimensional liver microtissues. Mol Cell Proteomics. 2015;14(5):1400-10.
14. UniProt C. The Universal Protein Resource (UniProt) in 2010. Nucleic Acids Res. 2010;38(Database issue):D142-8.
15. Zhou W, Quah ST, Verma CS, Liu Y, Lane DP, Brown CJ. Improved eIF4E binding peptides by phage display guided design: plasticity of interacting surfaces yield collective effects. PLoS One. 2012;7(10):e47235.
